# Novel aroylated phenylenediamine compounds enhance antimicrobial defense and maintain airway epithelial barrier integrity

**DOI:** 10.1038/s41598-019-43350-z

**Published:** 2019-05-08

**Authors:** Iwona T. Myszor, Zahida Parveen, Håkan Ottosson, Peter Bergman, Birgitta Agerberth, Roger Strömberg, Gudmundur H. Gudmundsson

**Affiliations:** 1 Biomedical Center, University of Iceland, Reykjavik, 101 Iceland; 2 Department of Biosciences and Nutrition, Karolinska Institutet, S-14183, Huddinge, Sweden; 3 Department of Laboratory Medicine, Clinical Microbiology, Karolinska Institutet, S-14186, Huddinge, Sweden

**Keywords:** Innate immunity, Cell signalling

## Abstract

Aroylated phenylenediamines (APDs) are novel inducers of innate immunity enhancing cathelicidin gene expression in human bronchial epithelial cell lines. Here we present two newly developed APDs and aimed at defining the response and signaling pathways for these compounds with reference to innate immunity and antimicrobial peptide (AMP) expression. Induction was initially defined with respect to dose and time and compared with the APD Entinostat (MS-275). The induction applies to several innate immunity effectors, indicating that APDs trigger a broad spectrum of antimicrobial responses. The bactericidal effect was shown in an infection model against *Pseudomonas aeruginosa* by estimating bacteria entering cells. Treatment with a selected APD counteracted *Pseudomonas* mediated disruption of epithelial integrity. This double action by inducing AMPs and enhancing epithelial integrity for one APD compound is unique and taken as a positive indication for host directed therapy (HDT). The APD effects are mediated through Signal transducer and activator of transcription 3 (STAT3) activation. Utilization of induced innate immunity to fight infections can reduce antibiotic usage, might be effective against multidrug resistant bacteria and is in line with improved stewardship in healthcare.

## Introduction

The airway epithelium plays a critical role in the first line of defense against respiratory pathogens. The pseudostratified bronchial epithelial layer is composed of undifferentiated basal cells and mature cells tightly linked by adherent and tight junctions providing a stringent barrier between the host milieu and the environment^1,2^. Protection is also provided by the mucus layer at the apical surface of the airway epithelium, which is a complex mixture of different glycoproteins and defense components such as antimicrobial peptides (AMPs). Mucociliary clearance by beating cilia drives movements of the mucus layer for the removal of particles and microbes^1,3–5^. Multiple human AMPs have been identified, the major two families include the defensins (α- and β-defensins) and the cathelicidin-family with one dominant peptide LL-37^6–8^. AMPs on the airway epithelial surfaces are expressed constitutively and can be induced by activation of pattern recognition receptors (PRRs) on the epithelial cells, such as transmembrane Toll-like receptors and intracellular NOD-like receptors. Cytokines can also affect the AMPs expression, as confirmed for IL-17 and IL-22^1,9^. The crucial role of AMPs in the first line of defense against respiratory pathogens was confirmed by using β-defensin-1 and cathelicidin deficient mice that were more susceptible to infections^10,11^. Besides broad-spectrum direct microbicidal activity, AMPs display immunomodulatory functions such as chemotaxis^6,12^. Similar to the defensins, LL-37 can also be immunomodulatory, stimulating airway epithelial cell proliferation and wound healing^13,14^. Recently, it was shown that LL-37 activates autophagy and promotes killing of intracellular *M. tuberculosis*^15,16^.

Human AMPs can be regarded as endogenous antibiotics because of their broad antimicrobial activity^14,17^. AMPs are important in host - pathogen interactions and could be utilized to fight infections, potentially including those caused by antibiotic resistant bacteria. Multidrug-resistant (MDR) pathogens are a serious threat for the society and healthcare as exemplified by growing number of infections with the ESKAPE pathogens (*Enterococcus faecium*, *Staphylococcus aureus*, *Klebsiella pneumoniae*, *Acinetobacter baumanni*, *Pseudomonas aeruginosa* and *Enterobacter spp*.), where some of these bacteria are opportunistic pathogens^18,19^. Specifically *P. aeruginosa* and *S. aureus* are pathogens causing respiratory tract infections that can be life-threatening for immunocompromised patients especially for those suffering from cystic fibrosis^20^. Therefore alternative therapies to treat infections are urgently needed^18,19^. The induction of endogenous AMPs could be an effective way of treating infections because many MDR strains are susceptible to different AMPs. Several different compounds inducing expression of AMPs to boost innate immunity have been shown effective in animal models and clinical trials for treatment of infectious diseases, e.g. pulmonary tuberculosis^21,22^. Vitamin D3 is a direct inducer of the *CAMP* gene expression, the gene encoding the antimicrobial peptide LL-37^23–25^. Another potent inducer is phenylbutyrate (PBA), a short chain fatty acid derivative and also a histone deacetylase inhibitor (HDACi)^26^. Interestingly, PBA treatment of *Shigella*-infected rabbits resulted in clearance of *Shigella* infection and counteracted the suppression of rabbit cathelicidin (CAP-18) in the gut and lung epithelium^27^. However, PBA has a fast turnover and is converted into phenylacetate by β-oxidation^28^, therefore high doses of PBA are needed to induce AMPs expression *in vitro* and *in vivo*. Additional potent *CAMP* gene inducers described recently are Entinostat and derivatives designated aroylated phenylenediamines (APDs)^29,30^. It has been shown that Entinostat stimulates *CAMP* gene expression via activation of STAT3 and HIF-1α transcription factors in human colonic epithelial cells^29^. Moreover, oral treatment of *Shigella*- and *Vibrio cholera-* infected rabbits with Entinostat improved their survival and restored production of the rabbit cathelicidin CAP-18 in gut epithelial surfaces^30,31^. Entinostat is an HDACi undergoing clinical trials as adjunctive cancer therapy^32^. However, Entinostat has a documented cytotoxicity^33,34^.

In this study we tested if new APDs, designated HO53 and HO56 could stimulate innate immunity responses in airway epithelial cells by enhancing the expression of endogenous AMPs and if that response was effective against the respiratory pathogen *Pseudomonas aeruginosa* PAO1 strain. We used bronchial epithelial cell lines, exhibiting a basal-like character and with the ability to differentiate towards polarized bronchial epithelium during air-liquid interface culture (ALI). In human bronchial epithelial cell lines, the new APDs markedly induced expression of the *CAMP* gene (encoding cathelicidin pro-LL-37/LL-37) both in monolayer and in ALI. The *CAMP* gene served as the reference, but also induction of other innate immunity genes involved in the defense against infections was observed. In the infection model with pretreatment of bronchial epithelial cells with the APDs significantly reduced the number of intracellular bacteria without exhibiting direct antibiotic properties. We could also demonstrate that treatment with one APD (HO53) of ALI cells counteracted the disruptive effect of *P. aeruginosa* conditioned medium by maintaining the epithelial barrier integrity. Utilizing a specific inhibitor, we showed that STAT3 transcription factor was involved in the HO53 mediated *CAMP* induction. Taken together, the current study might open up possibilities for using APDs as novel innate immunity modulators for host directed therapy (HDT) of infectious diseases.

## Results

### HO53 and HO56 induce *CAMP* gene expression in bronchial epithelial cell lines (BCi and VA10)

**Figure 1:**
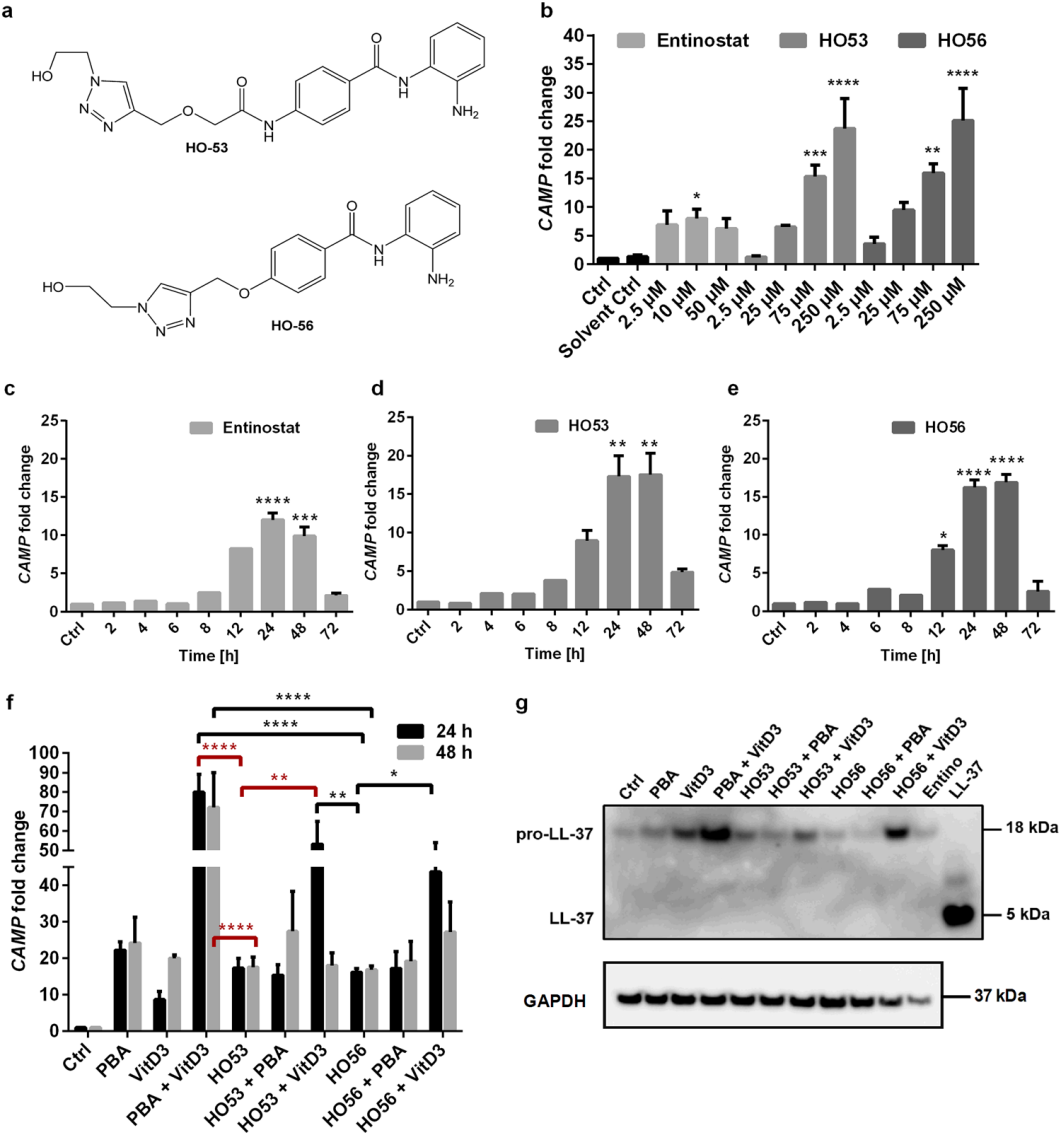
Enhanced induction of the *CAMP* gene by HO53 and HO56 in BCi cells. (**a**) Structure of the two novel aroylated phenylenediamine (APD) compounds investigated in this study, HO53 and HO56. (**b**) Induction of the *CAMP* gene with increasing concentration of Entinostat, HO53 and HO56 in BCi cells after 24 h post treatment. DMSO (final concentration lower than 1%) was used as a solvent control (Solvent Ctrl). Each bar represents mean value of 3 independent experiments ± SEM; statistical significance was calculated in comparison to control cells (Ctrl) using one-way ANOVA with Dunnett’s multiple comparisons test. Time-dependent *CAMP* induction by (**c**) Entinostat (10 μM), (**d**) HO53 (75 μM) and (**e**) HO56 (75 μM) in BCi cells, respectively. Bars for 2–8 h (for HO53 and HO56) and bars for 2–12 h (for Entinostat) represent values from two technical repeats. Bars for 12–72 h (for HO53 and HO56) and bars for 24–72 h (for Entinostat) are mean values of 3 independent experiments ± SEM; statistical significance was calculated in comparison to control cells (Ctrl) using one-way ANOVA with Sidak’s multiple comparisons test. (**f**) Cooperation of HO53 and HO56 (both at 75 μM) with sodium 4-phenylbutyrate (PBA; 2 mM) or 1α,25-dihyroxyvitamin D3 (VitD3; 100 nM) for the induction of the *CAMP* gene at 24 h and 48 h. Each bar represents mean value of 3 independent experiments ± SEM; statistical significance was calculated in comparison to HO53 (red) and HO56 (black) using two-way ANOVA with Dunnett’s multiple comparisons test. (**g**) Concentrated culture medium after 24 h stimulation was used for Western blot analyses using monoclonal antibody against LL-37. Positive control included 2 ng of synthetic LL-37 peptide. GAPDH analyzed in cell lysates was used as a loading control. The representative Western blot is one of 3 independent experiments. The full-length blots are presented in Supplementary Figure S10a. For all qRT-PCR experiments the *CAMP* gene expression was normalized to *TUBB* (tubulin-β) reference gene and presented as fold change of the expression compared to control cells (Ctrl) set as 1. Symbols for statistical analysis indicate p-values *p < 0.05, **p < 0.01, ***p < 0.001, ****p < 0.0001.

Entinostat has been confirmed as a potent inducer of AMPs, with effects against bacterial infections in animal models^30,31^, but is known to possess cytotoxic properties^33,34^ and has limited solubility in aqueous solutions. Based on the structure activity relations found in the first studies on APDs^30^, we started to optimize the AMP-inducing aroylated phenylenediamines (APDs) by designing and synthesizing new alternative compounds. The criterion was to reduce toxicity, while retaining efficient induction of AMPs and the design was based on making more hydrophilic APDs. Using the previously described luciferase reporter HT29 colonic cell line for expression-analysis of the antimicrobial peptide LL-37^35^, we identified, among the novel APDs, HO53 and HO56 (Fig. 1a; Supplementary Figs S1 and S2; Supplementary Methods) as interesting AMP-inducers with high activity but reduced toxicity.

The present study is focused on the effect of the two new compounds HO53 and HO56 (Fig. 1a) and with Entinostat as comparison. HO53 and HO56 enhanced *CAMP* gene expression in BCi-NS1.1 cells (BCi) at 24 h post treatment in a dose dependent way (Fig. 1b; Supplementary Fig. S3b and S3c). Entinostat also induced *CAMP* gene expression in BCi cells at 24 h but in contrast to HO53 and HO56, the induction was not dose dependent (Fig. 1b; Supplementary Fig. S3a). A similar pattern of induction with HO53 and HO56 was observed for the bronchial epithelial cell line VA10 (Supplementary Fig. S4). A broad range of HO53 and HO56 concentrations (2.5–250 µM) (Supplementary Fig. S3b and S3c) was tested and for further experiments we selected 75 µM for both compounds. These concentrations represented a low dose that significantly induced *CAMP* gene expression and had low effect on cytotoxicity and proliferation of BCi cells as compared to Entinostat, but comparable to PBA (Supplementary Fig. S5a and S5b). We used lower concentrations of Entinostat (2.5–50 µM) to keep the DMSO (solvent) concentration in the cell culture medium lower than 1% (v/v). However, there was no difference in the *CAMP* gene induction between the various concentrations of Entinostat in BCi cells and therefore we decided to use 10 µM in following experiments because of low cytotoxicity (Supplementary Fig. S5).

Next, we monitored *CAMP* gene expression over time (0–72 h) with the selected concentration of Entinostat (10 µM), HO53 and HO56 (both 75 µM) (Fig. 1c–e). Notably, the *CAMP* gene expression was increased significantly after 12 h and 24 h of treatment with HO56 and HO53, respectively and reached maximum expression at 24 h that was maintained up to 48 h post- treatment, but declined at 72 h. A similar effect was observed for Entinostat, where the maximal fold induction was after 24 h and lasted until 48 h, but declined at 72 h. Furthermore, we tested cooperation of HO53 and HO56 with the known *CAMP* gene inducers PBA (2 mM) and vitamin D3 (1α,25-dihydroxyvitamin D3; 100 nM) (Fig. 1f). We observed a synergistic effect between vitamin D3 and the two compounds at 24 h. However, no cooperation was observed upon treatment with PBA and HO53 or HO56 (Fig. 1f). Consistent with these findings, the synergistic effect after co-treatment with vitamin D3 was also reflected at the protein level by Western blot analyzes, where higher amount of pro-LL-37 was secreted to the cell culture medium (Fig. 1g). HO53 and HO56 separately and in combination with PBA did not affect the pro-LL-37 secretion. Taken together, our findings showed that HO53 and HO56 are novel, low toxic inducers of the *CAMP* gene expression in bronchial epithelium, working in synergy with vitamin D3.

### HO53 and HO56 induce several antimicrobial effectors in a bronchial epithelial cell line (BCi)

**Figure 2:**
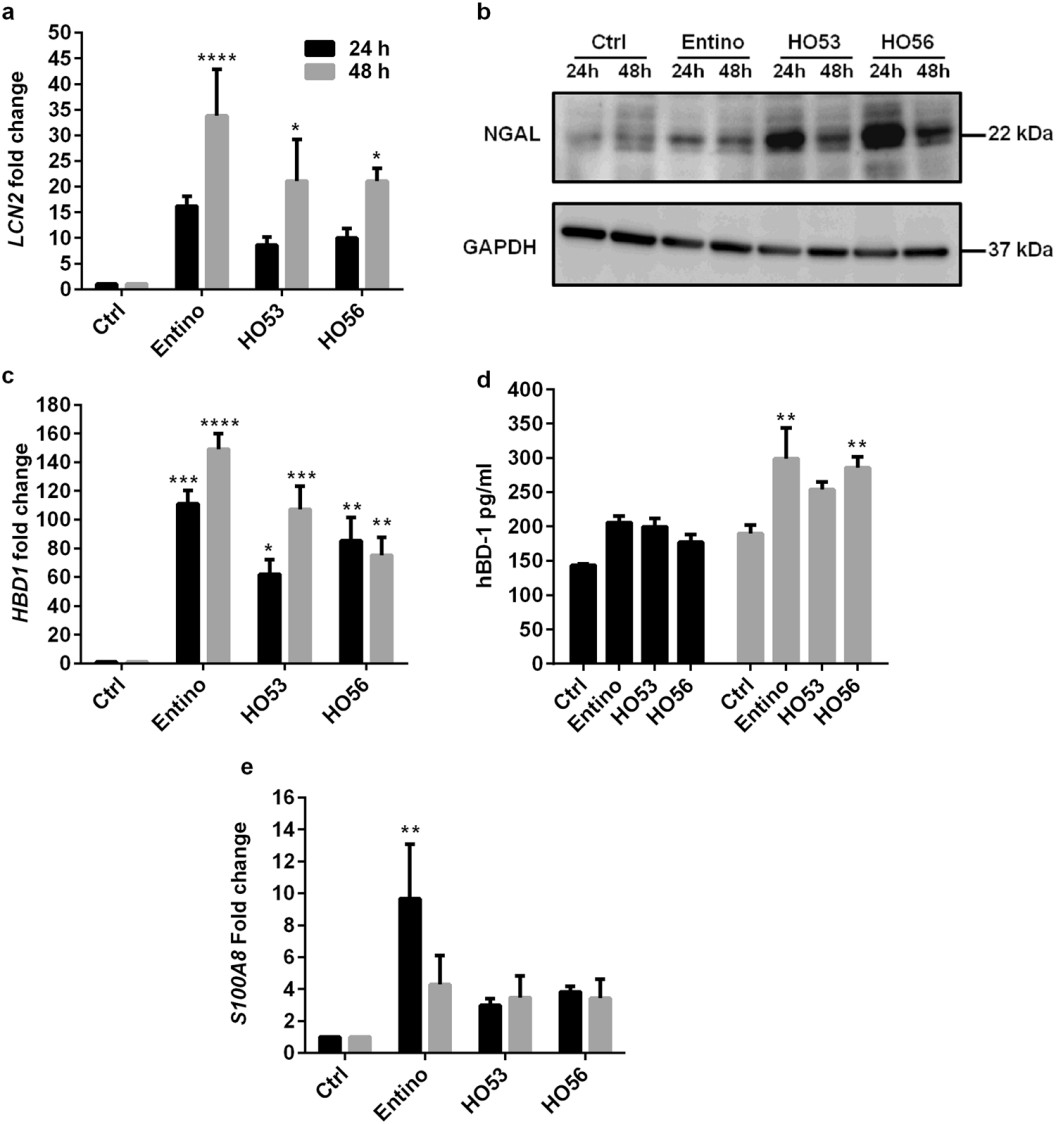
Enhanced expression of antimicrobial effectors by HO53 and HO56 stimulation in BCi cells. Samples were collected after 24 h (black bars) and 48 h (grey bars) post stimulation with Entinostat (10 μM), HO53 (75 μM) and HO56 (75 μM). Followed by expression analysis at mRNA and protein level by qRT-PCR and ELISA/Western blot, respectively. (**a**) Expression of *LCN2* (lipocalin 2) at mRNA and (**b**) protein (lipocalin 2, NGAL) level in cell lysates. The representative Western blot is selected from one of the 3 independent experiments. GAPDH was used as a loading control. Full-length blots are presented in Supplementary Figure S10b. (**c**) Fold change of *HBD1* (human β-defensin 1) mRNA in comparison to control (Ctrl) and (**d**) secretion of hBD-1 peptide measured by ELISA in cell culture supernatants. (**e**) *S100A8* expression was analyzed by qRT-PCR. *TUBB* (tubulin-β) was the reference gene in qRT-PCR. Each bar represents mean value of 3 independent experiments ± SEM; statistical significance was calculated in comparison to the control group using two-way ANOVA with Dunnett’s multiple comparisons test; *p < 0.05, **p < 0.01, ***p < 0.001, ****p < 0.0001. Significant changes are highlighted.

Further, we analyzed expression of additional antimicrobial proteins/peptides. *LCN2* encoding lipocalin 2, a siderophore binding protein that inhibits bacterial growth in iron deficient environment^36^. After 48 h stimulation with Entinostat, HO53 and HO56, the expression of *LCN2* at mRNA level was significantly increased (approximately 20–30 times) (Fig. 2a). The analyses at protein level revealed low induction of lipocalin 2 (also called Neutrophil gelatinase-associated lipocalin, NGAL) by Entinostat at 24 and 48 h and the representative relative NGAL/GAPDH ratio normalized to control was 1.85 and 1.45, respectively (Fig. 2b and Supplementary Table S2). In contrast to Entinostat, the induction of lipocalin 2 with HO53 and HO56 was prominent at 24 h (the representative relative NGAL/GAPDH ratio normalized to control was 6.56 and 6.98 respectively) and decreased at 48 h (2.24 and 2.23 for HO53 and HO56, respectively) (Fig. 2b and Supplementary Table S2). *HBD1* encoding the human β-defensin-1 antimicrobial peptide^37^ was significantly induced by Entinostat, HO53 and HO56 at mRNA level (Fig. 2c). Significant induction of human β-defensin-1 antimicrobial peptide at the protein level analyzed by ELISA was observed at 48 h (Fig. 2d). In addition, the *S100A8* gene encoding a unit of calprotectin antimicrobial protein^38^ was significantly induced by Entinostat (approximately 10 times) after 24 h stimulation, whereas induction with HO53 and HO56 was not significant (Fig. 2e). In contrast, the expression of lysozyme (*LYZ*), lactoferrin (*LTF*) and β-defensin 2 (*HBD2)* at mRNA levels was not detected in monolayer BCi cells. In summary the APD compounds affected multiple innate antimicrobial effectors in bronchial undifferentiated epithelial cells.

### HO53 and HO56 affect cytokine profiles in bronchial epithelial cell line (BCi)

**Figure 3:**
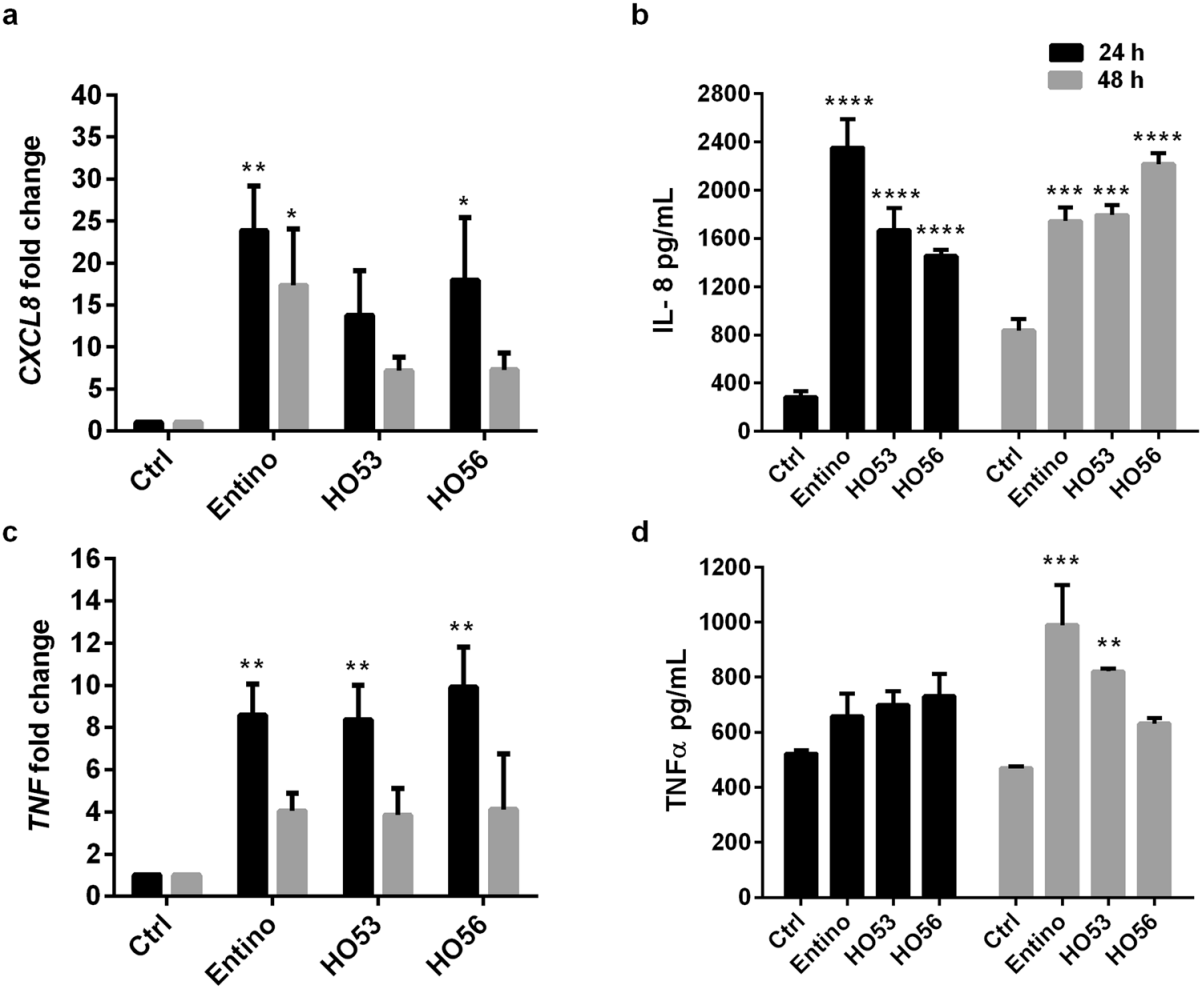
HO53 and HO56 alter expression of cytokines in BCi cells. Expression of (**a**) *CXCL8* and (**c**) *TNF* at mRNA level was analyzed by qRT-PCR and normalized to *TUBB* (tubulin-β) reference gene. The secretion of the proteins (**b**) IL8 (CXCL8) and (**d**) TNFα was evaluated by ELISA in corresponding cell culture medium. Analysis was performed at 24 h (black bars) and 48 h (grey bars) after stimulation with Entinostat (10 μM), HO53 (75 μM) and HO56 (75 μM). For all experiment each bar represents mean value of 3 independent experiments ± SEM; statistical significance was calculated in comparison to the untreated control cells (Ctrl) using two-way ANOVA with Dunnett’s multiple comparisons test; *p < 0.05, **p < 0.01, ***p < 0.001, ****p < 0.0001. Significant changes are highlighted.

We also evaluated the effect of Entinostat, HO53 and HO56 on the induction of the pro-inflammatory mediators IL-8 (CXCL8) and TNFα. Upon induction with Entinostat and HO56 a significant induction of mRNA for IL-8 was observed (Fig. 3a). However, at the protein level significantly increased secretion of IL-8 in cell supernatants was observed for the three inducing compounds (Fig. 3b). Induction of TNFα mRNA expression for three inducing compounds was observed only at 24 h (Fig. 3c) but at the protein level significant increased secretion was detected only in cell supernatants from Entinostat and HO53 stimulated culture at 48 h (Fig. 3d). In summary the APD compounds Entinostat, HO53 and HO56 affected the expression and release of specific cytokines and chemokines, indicating potent enhancement of innate immunity defenses in bronchial epithelial cells.

### HO53 and HO56 enhance antibacterial activity of the human bronchial epithelial cells (BCi)

**Figure 4:**
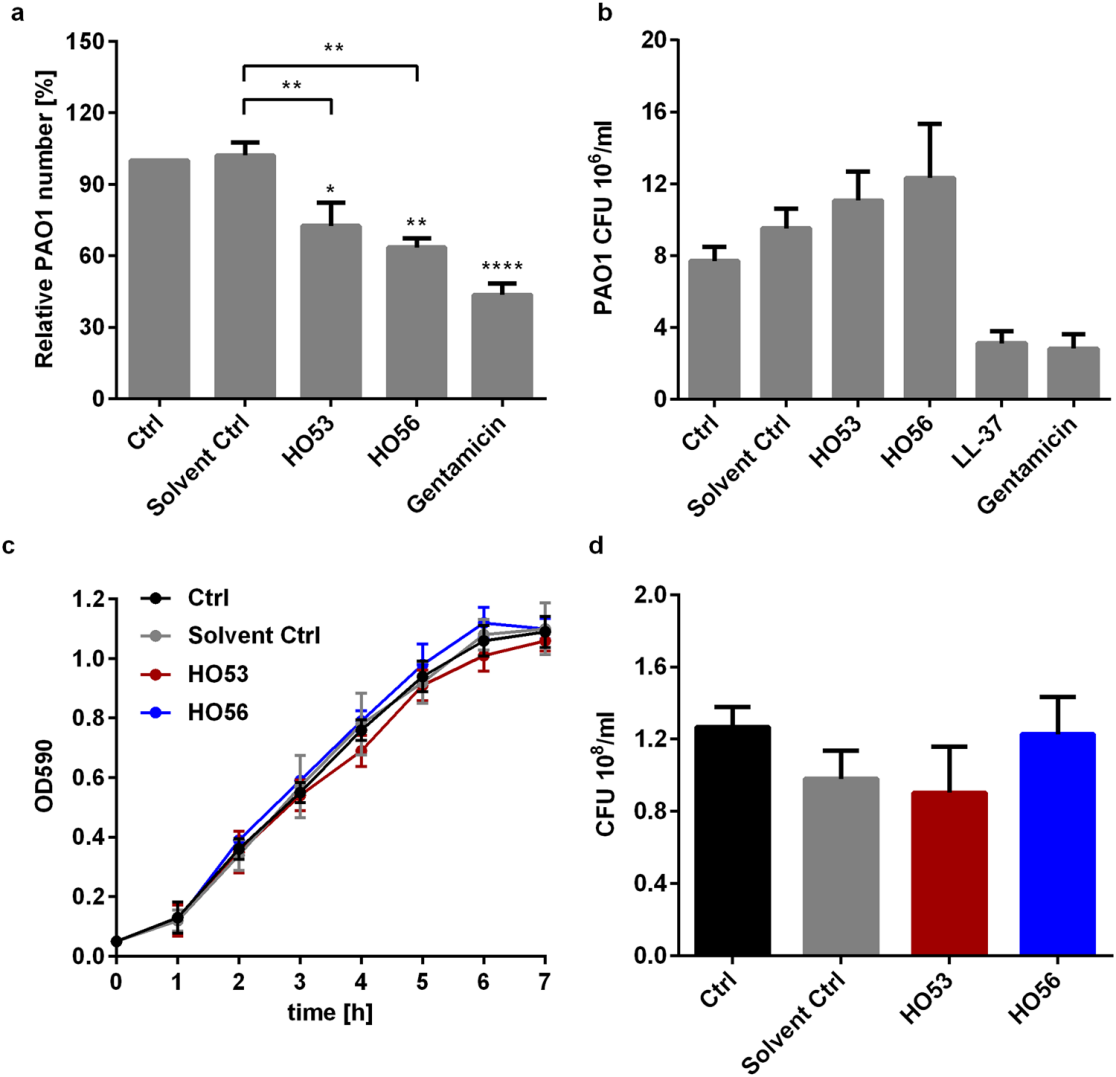
HO53 and HO56 enhance antimicrobial response in BCi cells but are not directly bactericidal. (**a**) Effect of APDs treatment on *Pseudomonas aeruginosa* PAO1 invasion of BCi cells. Cells were stimulated with HO53 and HO56 (both at 75 µM) for 24 h, then infected with PAO1 for 1 h and the remaining extracellular bacteria were eliminated with gentamicin (100 µg/ml) treatment for 20 min. Intracellular bacteria are presented as a percentage of colony forming units (CFU) from untreated cells (Ctrl) showed as relative PAO1 number [%]. Gentamicin at low concentration (0.5 µg/ml) was used as a positive control. (**b**) The direct effect of the two APDs on PAO1 in the cell culture medium (BEGM) in absence of BCi cells, presented as CFU after 1 h direct exposure. Gentamicin at low concentration (1 µg/ml) and synthetic LL-37 peptide (1 µg/ml) were used as positive controls. (**c**) *Pseudomonas aeruginosa* PAO1 growth and **d**) viability after 2 h co-incubation with the two APDs in Luria Bertani (LB) medium. Independent experiments of 3 ± SEM, for (**a**,**b** and **d**) statistical significance was calculated using one-way ANOVA with Dunnett’s multiple comparisons test while comparing to untreated group (Ctrl) or with Sidak’s multiple comparisons test while comparing to solvent control group. For (**c**) statistical significance was calculated using two-way ANOVA with Dunnett’s multiple comparisons test, *p < 0.05, **p < 0.01, ****p < 0.0001. Significant changes are highlighted.

We observed that HO53 and HO56 treatment enhanced production of AMPs and other innate immunity factors in human bronchial epithelial cells. Therefore, we tested if HO53 and HO56 treatment was effective in inducing a functional antimicrobial response in BCi cells against the respiratory pathogen *Pseudomonas aeruginosa* strain PAO1 (Fig. 4). BCi cells were treated for 24 h with HO53 and HO56, then infected for 1 h with multiplicity of infection (MOI) ~40, remaining extracellular bacteria were eliminated by treatment with gentamicin and the number of intracellular bacteria were enumerated as colony forming units (CFU). The number of intracellular PAO1 was significantly lower after HO53 (~75%) and HO56 (~60%) treatment in comparison to the PAO1 number in untreated control cells (100%; equal to ~1.0 × 10^4^ intracellular PAO1) (Fig. 4a). The treatment with low doses of gentamicin (0.5 µg/ml), a cell impermeable antibiotic, served as a positive control and reduced PAO1 entry to the cells about 50%. We performed an analogous experiment to the infection assay but in the absence of the BCi cells (Fig. 4b). After 1 h direct exposure of PAO1 to HO53 and HO56 in the cell culture medium, there was no significant differences in CFU counts (Fig. 4b). Furthermore, HO53 and HO56 did not inhibit PAO1 growth in Luria Bertani (LB) medium over time and did not kill bacteria after 2 h of direct exposure (Fig. 4c,d). To verify if the reduction of the intracellular PAO1 number was caused by induced antimicrobial polypeptides, we excluded additional antimicrobial effector systems that are involved in the defense of the epithelial surfaces, by analyzing the production of reactive oxygen species (ROS) (Supplementary Fig. S6a) and nitric oxide (NO) (Supplementary Fig. S6b, S6c)^39,40^. These findings indicated that the reduction of intracellular bacterial number was due to induced antimicrobial responses in human bronchial epithelial cells, but not any direct activity of the two APD compounds.

### HO53 and HO56 induce several innate immunity genes in polarized bronchial epithelial cells

**Figure 5:**
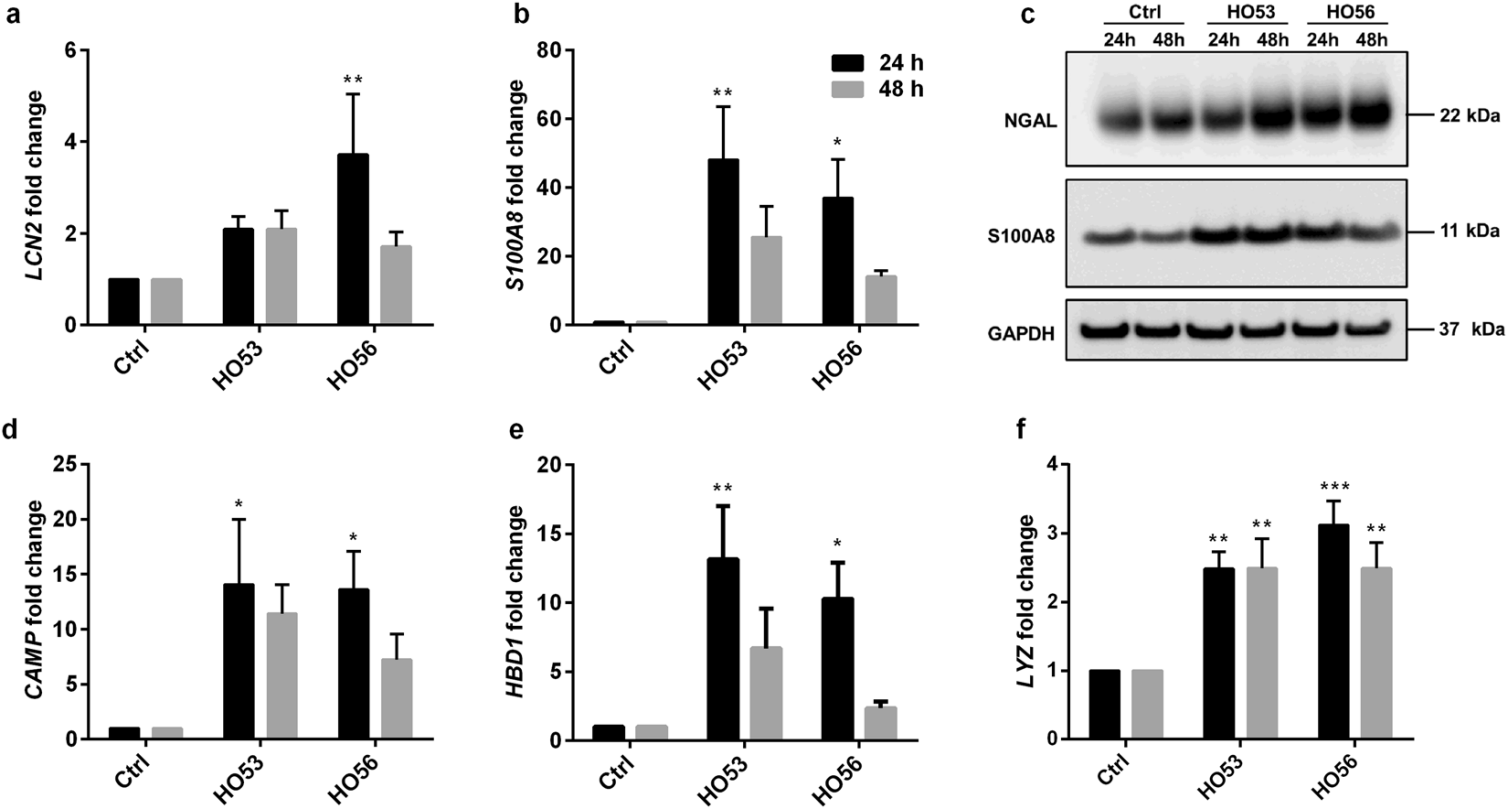
HO53 and HO56 enhance induction of additional innate immunity genes including cytokines in ALI differentiated BCi cells. Differentiated cells were used for experiments when TEER value reached approx. ≥ 1000 Ω × cm^2^. Expression at mRNA level of (**a**) *LCN2*, (**b**) *S100A8* and (**c**) subsequent protein level expression of NGAL and S100A8 analyzed by Western blot. GAPDH was used as loading control. The Western blot analysis was performed n = 2 with similar results. Full-length blots are presented in Supplementary Figure S10c. The mRNA level of (**d**) *CAMP*, (**e**) *HBD1*, (**f**) *LYZ* after 24 h (black bars) and 48 h (grey bars) stimulation with HO53 and HO56 (both at 75 μM). Expression at mRNA level was analyzed by qRT-PCR and normalized to *TUBB* (tubulin-β) reference gene. Data is from n = 4 independent experiments ± SEM, statistical significance was calculated in comparison to untreated cells (Ctrl) using two-way ANOVA with Dunnett’s multiple comparisons test, *p < 0.05, **p < 0.01, ***p < 0.001. Significant changes are indicated.

In order to transfer our findings into a more clinically relevant context, we tested if HO53 and HO56 treatment was effective in stimulating antimicrobial responses in polarized mature epithelium, mimicking the human bronchi^41^, we analyzed expression of AMPs in differentiated BCi cells. The mRNA levels of *LCN2* and *S100A8* were significantly higher compared to untreated cells, especially after 24 h post-treatment with HO56 (~4 and ~40 fold, respectively) (Fig. 5a,b). We also observed increased protein levels of lipocalin 2 (NGAL) and S100A8 after 24 and 48 h of treatment with HO53 and HO56. However, induction of lipocalin 2 was more pronounced after 48 h (Fig. 5c). Further, we analyzed the effects of HO53 and HO56 treatment on the expression of the genes *CAMP*, *HBD1* and *LYZ* in differentiated BCi cells (ALI). The *CAMP* gene expression was on a similar level as the cells in monolayer (upregulated about 15-times) but that effect was not observed after 48 h of treatment with HO53 and HO56 (Fig. 5d). The enhanced expression of *HBD1* (Fig. 5e) and *LYZ* (Fig. 5f) upon treatment with HO53 and HO56 was significantly higher (~10 times and 3 times, respectively). Furthermore, the expression of pro-inflammatory cytokines/chemokines was not significantly upregulated, except the expression of *IL1B* after 24 h of treatment with HO53 (Supplementary Fig. S7d). In contrast to monolayer cells, the expression of *TNF*/TNFα and *CXCL8*/IL-8 was not significant (Supplementary Fig. S7). In summary, we observed a different induction profile of antimicrobial effectors and cytokines/chemokines for differentiated cells in comparison to monolayer cells.

### HO53 counteracts disruptive effect of *P. aeruginosa* conditioned medium on airway epithelial integrity

**Figure 6:**
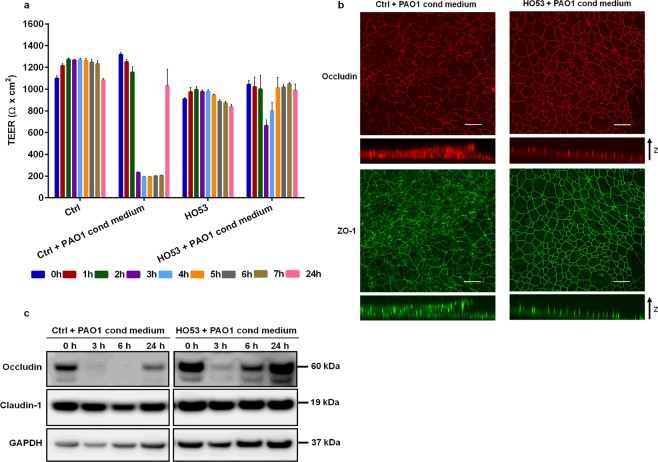
HO53 treatment counteracts the disruptive effect of *P. aeruginosa* PAO1 conditioned medium in ALI differentiated BCi cells. Differentiated BCi cells (trans epithelial electrical resistance, TEER ≥ 1000 Ω × cm^2^) were treated with 75 μM HO53 in the lower chamber of transwell insert for 3 days and challenged with PAO1 conditioned medium (PAO1 cond medium) applied on the apical surface of the cells for the next 24 h. (**a**) TEER measurement after every hour from 0 to 7 h and after 24 h post PAO1 culture medium challenge. Data shown is representative of two experiment and showing mean values of four independent ALI filters at each time point ± SEM. Samples for confocal microscopy and Western blot were collected at selected time points in order to analyze tight junctions (TJ) integrity. (**b**) Confocal images showing disruption of occludin (red) and ZO-1 (green) after 6 h post PAO1 culture medium challenge of HO53 treated cells as compared to control cells (Ctrl), *Bar* = 10 µm. (**c**) Western blot analyses of occludin and claudin-1 after 0, 3, 6 and 24 h exposure to PAO1 culture medium. GAPDH was used as a loading control. The full-length blot for occludin is presented in Supplementary Figure S9 and for claudin-1 and GAPDH in Supplementary Figure S10d.

Pathogens exhibit different strategies to evade the host epithelial surfaces. *P. aeruginosa* can disintegrate the junctions between epithelial cells and enter tissue through the paracellular space^42^. Another strategy is the production of virulence factors that can be injected into the host cells or secreted by bacteria to the environment, e.g. rhamnolipids^43^. We have shown that *Pseudomonas aeruginosa* PAO1 conditioned medium can disrupt epithelial barrier integrity^44^. Here we investigated if the treatment with HO53 can counteract the disruptive effect of PAO1 conditioned medium (Fig. 6). BCi cells differentiated in ALI culture were pretreated with HO53 for three consecutive days and challenged with PAO1 conditioned medium applied on the apical side of ALI culture for 24 h. Azithromycin was used as a positive control (Supplementary Fig. S8). After 3 h exposure of control BCi cells to PAO1 conditioned medium, trans-epithelial electrical resistance (TEER) decreased from ~1200 Ω × cm^2^ to ~200 Ω × cm^2^ and the disruptive effect was also observed after 6 h of exposure (Fig. 6a). The recovery of the tight junctions (TJs) integrity was noted after 24 h post challenge. In contrast to the control cells, the pretreatment of the ALI culture with HO53 did not lead to a pronounced TEER drop (~1000 Ω × cm^2^ to ~700 Ω × cm^2^) and the epithelial barrier integrity was restored after 5 h. To illustrate counteraction of TJ disruption, we performed confocal microscopy after 6 h of PAO1 conditioned medium challenge. Consistent with the TEER results, the disruption of zonula occludens (ZO-1) and occludin was observed in the control group, while HO53 treated group maintained TJs integrity (Fig. 6b). We further analyzed levels of the tight junction proteins occludin and claudin-1 at selected time points (0, 3, 6, 24 h) by Western blot analysis (Fig. 6c). The band corresponding to the main occludin isoform was faintly detected after 3 and 6 h of PAO1 conditioned medium challenge in the control group (the western blot analyses shows the occludin degradation/processing product, Supplementary Fig. S9) and did not reach the same expression level after 24 h. However, in the cells pretreated with HO53 occludin was not degraded. After 3 h of challenge the occludin level was reduced, while after 6 h the expression was restored. Notably, induction of occludin expression was detected upon HO53 treatment, whereas there was no change in claudin-1 level (Fig. 6c). In conclusion, HO53 counteracted disruptive effect of PAO1 conditioned medium on respiratory epithelium via a novel mechanism affecting tight junctions.

### STAT3 activation is required for HO53 mediated *CAMP* gene induction

**Figure 7:**
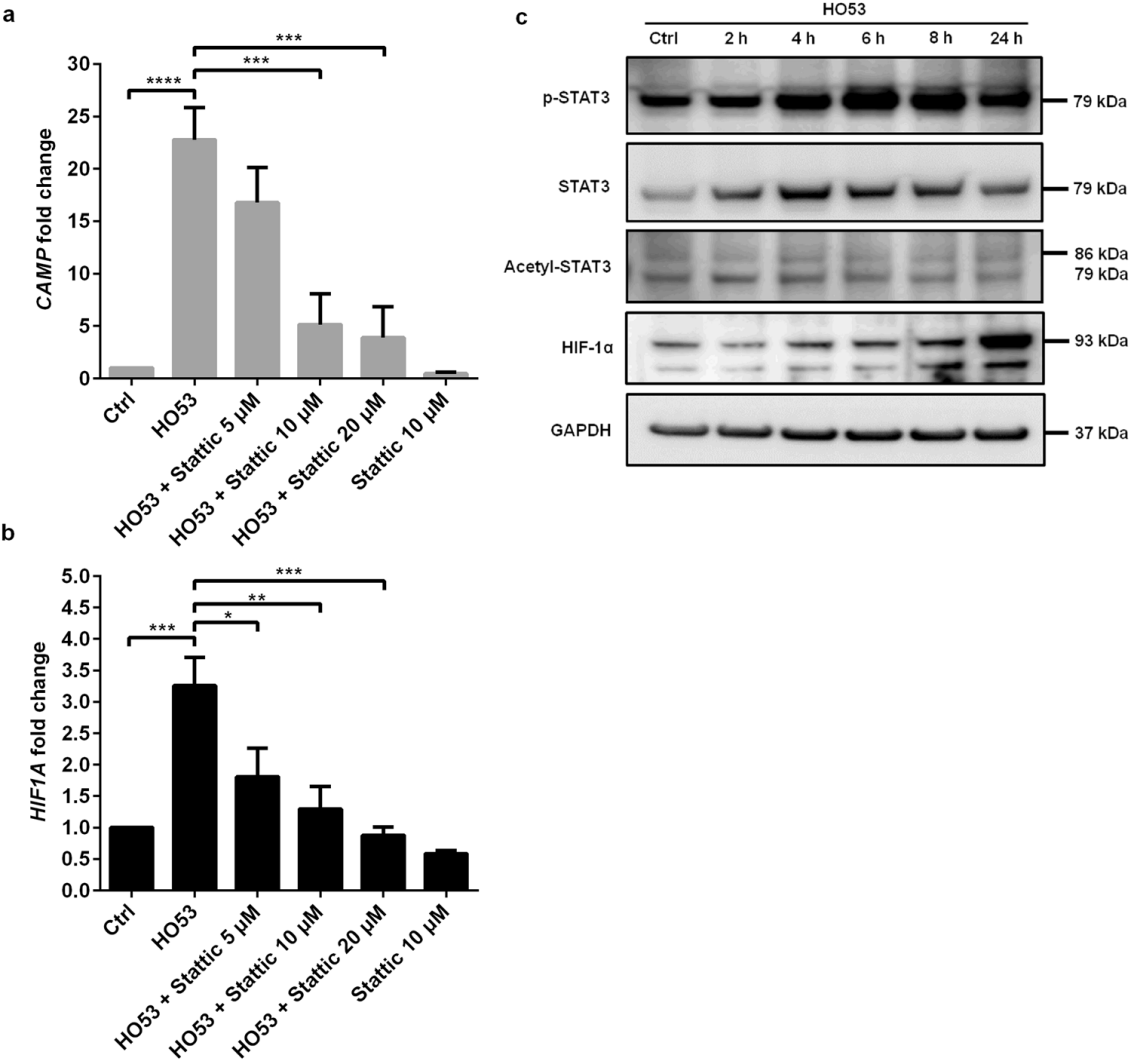
STAT3 and HIF-1α mediate *CAMP* gene induction by HO53 in BCi cells. The effect of the STAT3 inhibitor Stattic on (**a**) *CAMP* and (**b**) *HIF1A* expression in BCi cells. Cells were pretreated with 5, 10 and 20 µM of Stattic for 30 min and then stimulated with HO53 (75 µM) for 24 h. *CAMP* and *HIF1A* expression was analyzed by qRT-PCR and normalized to *TUBB* (tubulin-β) reference gene. The data (**a**,**b**) represent mean of n = 4 independent experiments ± SEM. Statistical significance for HO53 and Stattic 10 µM was calculated in comparison to control (Ctrl), while the inhibitory effect of Stattic refers to HO53 alone using one-way ANOVA with Sidak’s multiple comparisons test, *p < 0.05, **p < 0.01, ***p < 0.001, ****p < 0.0001. Significant alterations are highlighted. (**c**) Effect of HO53 (75 µM) treatment of BCi cells on STAT3 phosphorylation (p-STAT3 at Tyr705), total STAT3, acetylation (Acetyl-STAT3 at Lys685) and HIF-1α expression after 2, 4, 6, 8 and 24 h was analyzed by Western blot. GAPDH served as a loading control. The experiment was performed n = 2 with similar results. Full-length blots are presented in Supplementary Figure S10e.

It has been shown that Entinostat induces *CAMP* gene expression in gut epithelial cells *via* activation of STAT3 and HIF-1α transcription factors^29^. HO53 is structurally related to Entinostat and here we investigated if the molecular pathways involved in the HO53 induced *CAMP* gene expression in bronchial epithelial cells follows similar pathways as described for the colon epithelial cell line HT-29^29^. For that, we examined the role of STAT3 by using the STAT3 inhibitor Stattic (Fig. 7a,b). BCi cells were pretreated with increasing doses of the Stattic inhibitor (5 µM, 10 µM, and 20 µM) for 30 min followed by stimulation with HO53 for 24 h. We observed significant dose-dependent Stattic mediated decrease in *CAMP* gene expression (Fig. 7a). Transcription factor HIF-1α expression was also decreased in a dose-dependent manner as for the *CAMP* gene (Fig. 7b). Furthermore, we investigated if HO53 treatment resulted in enhanced STAT3 expression or post-translational modifications (Fig. 7c). The expression level of STAT3 and phosphorylated-STAT3 was increased in a time dependent manner after treatment with HO53. The level of the phosphorylated STAT3 increased after 4 h and reached the maximum after 6 h of treatment with HO53. We did not observe any difference in the acetylation at Lys685 of STAT3 after treatment with HO53. However, modifications of other lysine residues of STAT3 cannot be excluded. HIF-1α expression level was gradually elevated at 4, 6 and 8 h and at 24 h there was a prominent enhanced level of HIF-1α. In summary, STAT3 is most likely a central regulator of the *CAMP* gene induction by HO53 although other STAT3 modifications need to be evaluated to explain the detailed mechanism of the inducing effect of HO53.

## Discussion

An increasing number of infections caused by antibiotic resistant pathogens contribute to higher morbidity/mortality rate and generate high costs for the health care system^45^. Therefore, development of alternative strategies to conventional antibiotic therapy is urgently needed. Host directed therapy (HDT) based on inducing innate immunity by enhancing expression of endogenous antimicrobial components or counteracting pathogen-mediated suppression of first line defenses could be an alternative^27^. HDT could limit the selection of antibiotic resistant strains and might be used against multidrug-resistant (MDR) bacteria.

Development of novel stable compounds for the induction of innate immunity would be beneficial. To approach this aim, a reporter cell line with the *CAMP* gene fused to the luciferase gene was established for screening induction of the *CAMP* gene expression^35^. Screening of compound libraries and selected HDAC inhibitors resulted in the identification of Entinostat, working against *Shigella* and *Vibrio* infections in rabbits^30,31^. Recently, a similar strategy for identification of other innate immunity inducers was described based on the induction of the defensins genes *HBD2* and *HBD3*^46,47^. Another interesting approach was through the pattern recognition receptor NOD2 (Nucleotide Binding Oligomerization Domain Containing 2), when activation of this receptor by N-phosphonacetyl-L-aspartate (PALA), was identified as a potent inducer of innate immunity. Expression of *HBD2* and *CAMP* were induced by PALA and antimicrobial activity was demonstrated in skin explants against bacteria including methicillin-resistant *Staphylococcus aureus* (MRSA)^48^.

In the current study we describe that the novel APD compounds HO53 and HO56 induced a broad spectrum of antimicrobial effectors in bronchial epithelium. Both compounds are developed as AMP inducers with increased water solubility, lower cytotoxicity and reduced effects on cell proliferation as compared to Entinostat. We mainly used the BCi-NS1.1 (BCi) cell line^41^ but the induction of the *CAMP* gene expression was also confirmed in the VA10 cell line^49^. The gene induction profiles of HO53, HO56 and Entinostat were similar with reference to time but not with reference to the concentration. The dose studies utilizing BCi cells revealed a concentration dependent response for HO53 and HO56, but not for Entinostat (Fig. 1b; Supplementary Fig. S3) most likely due to higher cytotoxicity of the latter. Interestingly, when HO53 and HO56 were tested with the known innate immunity inducers vitamin D3 and PBA, a synergistic induction of the *CAMP* gene expression was noted with vitamin D3 but not with PBA. This suggests that APDs and PBA possibly act through the same signaling pathways. PBA and Entinostat are known HDAC inhibitors^28,32^, however today this activity is referred to as lysine deacetylases inhibition (KDACi) because apart from histones also cytoplasmic signaling pathway proteins are affected^50^. Indeed, the suggested mechanism of Entinostat activity included acetylation of the cytoplasmic signaling protein STAT3^29^. Therefore, possible KDAC inhibition by the novel APD compounds must be experimentally confirmed.

For both novel APDs we found induced antimicrobial activity in BCi cells against *Pseudomonas aeruginosa* (PAO1) and the disruptive effect of PAO1 on epithelial integrity was counteracted by HO53. This double effect of HO53 on the epithelial barrier might be potentially beneficial *in vivo* for fending off bacterial intruders and blocking bacterial translocation. Thus, HO53 would be an interesting candidate to treat and/or prevent infections. Interestingly, the antimicrobial activity was assessed against *Pseudomonas aeruginosa* that is prevalent in immune compromised lungs, including cystic fibrosis patients. The characteristics of the cellular response to treatment with HO53 suggest an effective HDT compound that might be beneficial for cystic fibrosis patients and used against MDR strains.

Induction of the *CAMP* gene expression served as a reference for the selection of HO53 and HO56 compounds. We selected additional genes encoding peptides/proteins with known defense functions at epithelial surfaces such as lipocalin 2, HBD1, S100A8, lysozyme and lactoferrin to verify whether the induced expression would mimic *in vivo* defenses. Significant induction of lipocalin 2, HBD1 and S100A8 expression was observed at mRNA and protein levels, indicating the APD compounds as powerful activators of a broad spectrum defense. Kinetics of mRNA and protein induction were not strictly correlated, where the most pronounced deviation was for *HBD1* expression with a fold change of mRNA from 50 to 100, while at protein level only approximately 2 times changed (Fig. 2c,d). Similar differences were also observed for lipocalin 2, indicating a regulation at translational level in the cells. However, the current overall expression profile shows a broad induction of multiple polypeptides. In addition, we estimated the influence of APDs on reactive oxygen species (ROS) and nitric oxide (NO) production. ROS was not affected but the expression of *NOS2* (encoding inducible nitric oxide synthase iNOS) was enhanced. However, this enhancement was not confirmed on effector level by the Griess method, measuring nitrate in solution probably due to low concentrations. The observed antimicrobial effect in our system is unlikely due to enhanced NO production. However, low induction of the iNOS protein may contribute to the antimicrobial effect *in vivo*. Thus, the main antibacterial activity in our model is most likely due to increased expression of antimicrobial proteins/peptides and could even be enhanced in the *in vivo* situation with complete processing of active components.

We analyzed the induction of different innate immunity effectors expression both in monolayer BCi cells and air liquid interphase (ALI), thereby comparing basal like cells with differentiated epithelial cells^41^. Several differences were observed: (1) the mRNA expression for lysozyme (*LYZ*) was not detected in monolayer but was inducible in differentiated cells (Fig. 5f), (2) induction of *S100A8* expression was more pronounced in ALI culture (S100A8 could not be detected in undifferentiated cells). (3) In contrast, the *HBD1* expression was more pronounced in undifferentiated cells. (4) The expression of pro-inflammatory effectors like, *TNF*/TNF-α and *CXCL8*/IL-8 was significantly enhanced in monolayer cells (Fig. 3) but not in ALI cultured cells, except *IL1B* expression (Supplementary Fig. S7). These differences indicate that the undifferentiated cells are more sensitive to external stimuli and trigger NF-kB regulated responses, which might be linked to their basal functions. The different responses can also reflect different transcription factors setup and chromatin accessibility in the basal cells versus polarized cells.

In the experiments for approaching molecular mechanism and epithelial integrity we selected HO53 because of lower cytotoxic effects on BCi cells, higher yields from the synthesis and better solubility than HO56. One suggested mechanism for induction of innate immunity by Entinostat included two steps: first, activation of STAT3 by acetylation and second, subsequent increase of HIF-1α^29^. Notably, the transcription factor HIF-1α has been confirmed important for transcription of innate immunity genes^51^. Because the new APDs are structurally related to Entinostat, we tested the effect of HO53 in relation to the STAT3 transcription factor using a specific inhibitor - Stattic. By blocking STAT3 we observed significant reduction of *CAMP* and *HIF1A* expression upon HO53 treatment. Furthermore, upon HO53 treatment there was a time dependent increase of the STAT3 protein and in particular a pronounced effect on the phosphorylation of STAT3, whereas the acetylation status at Lys685 was unchanged. Gradually increased expression of HIF-1α was detected with time after stimulation of the cells by HO53. In conclusion, STAT3 seems to be an important mediator of the APD response but more detailed studies on STAT3 modifications are needed that might define the cellular target of HO53.

In gut epithelia STAT3 mediated expression of occludin was shown to enhance tight junction function and prevent bacterial translocation^52^. HO53 treatment led to increased occludin expression (Fig. 6) that could be an explanation for limited disruption of tight junctions (TJs) caused by *Pseudomonas*. This type of counteraction was initially identified on polarized lung epithelium for the antibiotic azithromycin (AZM) by an unknown mechanism^44^. Here, we confirmed the effect by AZM (Supplementary Fig. S8), but interestingly the HO53 effect seemed to be more potent in rescuing the barrier integrity than AZM. Together all these effects of HO53 do not only underline the double action of the molecule but also highlights the importance of STAT3 in epithelial immunity^53,54^.

Our results warrant continuation in animal infection models and motivate pharmacodynamics and pharmacokinetic studies. Usage of the APDs could re-establish the niche for the natural microbiota and avoid selection of resistant bacteria in line with improvement of stewardship in healthcare.

## Materials and Methods

### Reagents and materials

Entinostat (SNDX-275), 1α,25-dihydroxyvitamin D3 (D1530), collagen from human placenta (C7521), Stattic inhibitor (S7947), LB broth (L3522), agar (05039-500G), gentamicin (G1914), methanol (34885-2.5L), menadione (M5625), the secondary antibodies conjugated with HRP for Western blotting (A5420 and A0545), DAPI (D9564) were purchased from Sigma. UltroserG (UG, 15950-017) was obtained from PALL Life Sciences and sodium 4-phenylbutyrate (2682) from Tocris Bioscience. Synthetic LL-37 peptide (SP-LL37) was bought from Innovagen and DMSO (sc-358801) from Santa Cruz. Azithromycin (Zitromax) of 500 mg was from Pfizer. The monoclonal anti-LL-37 antibody was generated by us and described in Yoshio *et al*.^55^. Anti-ZO-1 (#13663), anti-STAT3 (#9139), anti-acetyl-STAT3 (#2523), anti-phospho-STAT3 (#9145) and anti-GAPDH (#2118) antibodies were purchased from Cell Signaling Technology. Anti-lipocalin 2 (AF1757) antibody was obtained from R&D Systems, anti-HIF-1α (ab113642) antibody was from Abcam and anti-occludin (331500) and anti-claudin-1 (51-9000) antibodies from Thermo Scientific. Secondary antibodies for immunofluorescence staining were obtained from Thermo Scientific (A-11070 and A-11020). 2-Azidoethanol was synthesized according to a published procedure and distilled under reduced pressure^56^. All other synthesis reagents and solvents (analytical grade) were purchased from commercial sources and were used without further purification. The NMR spectra were collected on a Bruker DRX-400 spectrometer (400 MHz for 1H and 101 MHz for 13C) with the residual solvent signal as chemical shift reference. Mass spectra were recorded on a Micromass LCT (ESI-TOF) mass spectrometer.

### Cell cultures

The human bronchial epithelial cell line BCi-NS1.1 (BCi) immortalized with retrovirus expressing human telomerase (hTERT) was from dr Matthew S. Walters, Weill Cornell Medical College, New York NY, USA^41^. An E6/E7 viral oncogene immortalized human bronchial epithelial cell line VA10 has been described previously^49^. Both cell lines were cultured in Bronchial/Tracheal Epithelial cell growth medium (BEGM) (Cell Applications, 511A-500) supplemented with retinoic acid (Cell Applications, 511-RA) and Penicillin-Streptomycin ((20 U/ml, 20 µg/ml, respectively) (Life Technologies, 15140122)) at 37 °C and 5% CO_2_. The ALI (air-liquid interface) culture of BCi cells were maintained as described previously^41^. Cells in monolayer were treated with HO53 and HO56 by direct addition to the culture medium. Differentiated BCi cells were used for experiments when TEER value of ≥1000 Ω × cm^2^ was reached and then HO53 and HO56 were added to the lower chamber for indicated period of time. Azithromycin was used as a control.

### Bacterial culture

The overnight culture of *Pseudomonas aeruginosa* PAO1 strain was diluted in LB medium to OD_590_ = 0.05 and cultured at 37 °C with 180 rpm shaking until the bacterial subculture reached the mid-log phase.

### RNA analysis

Total RNA was extracted using Nucleo Spin RNA kit (Machinery-Nagel, 740955.50). Total RNA of 1 µg was used to synthesize complementary DNA (cDNA) according to the manufacture’s recommendations using the High Capacity cDNA reverse transcriptase kit (Applied Biosystems, 4368814). One µl of cDNA, 5 µl of PowerUp SYBR Green Master Mix (Applied Biosystems, A25742) and 0.5 µM primers enlisted in Supplementary Table S1 were used for qRT-PCRs. The qRT-PCRs were performed using LG 7500 Real Time PCR System (Applied Biosystems) with the following cycling conditions: (1) holding stage: 95 °C for 10 min, followed by 40 cycles of (2) denatured stage: 95 °C for 15 s and (3) annealed/extended stage: 60 °C for 1 min. The 2^(−ΔΔCT)^ Livak method was utilized for calculating fold differences over untreated control^57^.

### Immunoblotting

Cells were washed with PBS and lysed in RIPA lysis buffer (Santa Cruz, sc-364162) supplemented with Halt Protease Inhibitor Cocktail (Thermo Scientific, 87786) on ice for 30 min. The cell culture media in the volume of 2 ml were concentrated as it has been described earlier^58^. The concentrated culture media or 10–30 µg of total protein content was separated using NuPAGE 4–12% Bis–Tris gradient gel (Life Technologies, NP0323), NuPAGE MES SDS Running Buffer (Life Technologies, NP0002) and the running conditions were 120 V and 275 mA. The proteins were transferred on to a PVDF membrane (0.2 µm pores) using XCell II^TM^ Blot Module (Invitrogen, EI9051) and the membrane was blocked with 10% skimmed milk or 5% BSA in TBS-T buffer (50 mM Tris, 150 mM NaCl, 0.1% Tween-20) for 1 h at room temperature. Then, the membrane was incubated with primary antibodies overnight at 4 °C using a dilution recommended by the manufacturer’s protocol. After washing with TBS-T buffer the membrane was incubated with horse radish peroxidase (HRP) conjugated secondary antibodies (1:10 000 dilution) in 5% skimmed milk or 5% BSA in TBS-T for at least 1.5 h at room temperature. Immunoblots were developed using Pierce ECL Plus Western blotting substrate (Thermo Scientific, #34095) or Western blotting Luminol reagent (Santa Cruz, sc-2048) and ImageQuant LAS 4000 system (GE Healthcare). Quantification of the band intensity was performed using ImageJ software.

### ELISA

Sandwich enzyme-linked immunosorbent assays (ELISAs) were performed utilizing a human beta defensin-1 (hBD-1), interleukin 8 (CXCL8) and tumor necrosis factor alpha (TNFα) assay kit according to the manufacturer’s instructions (Peprotech, UK). The results are represented from three independent experiments.

### Cytotoxicity and cell viability assays

BCi cells (10,000) were seeded in 96-well plate chambers in 200 µl of BEGM. Once adhesion was verified, cells were incubated with different concentrations of Entinostat (2.5–50 µM), HO53 (2.5–250 µM) and HO56 (2.5–250 µM) along with the known *CAMP* inducer PBA (4 mM) for 24 h. Cytotoxicity and cell proliferation was determined using CytoTox 96® Non-Radioactive Cytotoxicity Assay (Promega, G1781) kit and Cell Proliferation Reagent WST-1 (Roche, 05015944001), respectively, according to the manufacturer’s instructions. The results presented were from three independent experiments for cytotoxicity and two independent experiments for cell proliferation.

### Antimicrobial assays

BCi cells in monolayer were treated for 24 h with APDs or low dose of gentamycin (0.5 µg/ml) used as a positive control and then infected with PAO1 using MOI ~40 for 1 h. Cells were then washed 3 times with PBS and incubated for 20 min with medium containing 100 µg/ml gentamicin (bacterial killing concentration). Cells were washed again, lysed in 0.1% Triton X-100/H_2_O (v/v) for 5 min, serially diluted in PBS and plated on LB agar plates. After overnight incubation at 37 °C, the PAO1 CFU (colony forming units) were enumerated.

### ROS detection

The H_2_O_2_ level in the cell culture medium after 24 h of treatment with HO53 and HO56 (both at 75 µM) was measured using ROS-Glo^TM^ H_2_O_2_ assay (Promega, G8820) according to the manufacture’s protocol.

### Agarose gel electrophoresis

Semi-quantitative PCR products were separated on 1.5% agarose gel containing ethidium bromide (0.5 µg/ml). Electrophoresis was run at 80 V for 30 min using 1x TAE (Tris-acetate-EDTA) buffer. β-tubulin was used as a control for the quantification of the band intensity using ImageJ software.

### Preparation of PAO1 conditioned medium and challenge of differentiated BCi cells

Wild-type (WT) *P. aeruginosa* strain PAO1 was used to prepare bacterial conditioned medium. Shortly, the bacteria were cultured in Dulbecco’s Modified Eagle Medium F-12 Nutrient Mixture (DMEM/F12) + 2% UltroserG (UG) at 30 °C and shaking was at 180 rpm for 5 days. Bacterial culture supernatants were collected, vortexed thoroughly, centrifuged, and filtered through 0.22 mm pore-size filter (GE Healthcare and Life Science, Whatman, Germany). BCi cells were cultured at the ALI for 3 weeks with medium changed every 2–3 days to get differentiated cells, followed by placing HO53 (75 µM diluted in DMEM/F12 + 2% UG) in the basal chamber of the transwell insert for 3 consecutive days, while Azithromycin was used as positive control as described previously^44^. Next, differentiated cells were challenged with PAO1 conditioned medium and TEER was measured after every hour. Samples for Western blot and confocal microscopy analyzes were collected at selected time points.

### Immunofluorescence staining and confocal microscopy

BCi cells growing on ALI filters were fixed using chilled methanol at 4 °C overnight followed by chilled acetone. Briefly, staining was done as follows: filters were hydrated with IF buffer (PBS + 0.3% Triton X-100), blocked with 10% FBS, washed and incubated with a primary antibody overnight at 4 °C. The following primary antibodies were used: rabbit anti-occludin and mouse anti-ZO-1. Next day the filter was washed and incubated with a secondary antibody for 2 h. For immunofluorescence staining, isotype-specific Alexa Fluor secondary antibody conjugates were used and DAPI was used to stain nuclei. The filter was then washed with IF buffer and finally rinsed with water. Cell culture transwell filters were mounted in Fluoromount™ Aqueous Mounting Medium (F4680-Sigma) and coverslips were placed over the filters. Images were captured using Olympus fluoview Fv1200 confocal microscope at 30x magnification. Z-scans were performed by taking series of images at the same location with fixed focal intervals.

### Statistical analysis

Results are presented as mean ± standard error of mean (SEM) from at least three independent experiments, otherwise it is indicated in the figure legends. One- or two-way ANOVA with post-hoc Dunnett’s or Sidak’s multiple comparisons test were used to determine significance of the data. *p* values are included in the figure legends. The statistical analysis were performed with GraphPad Prism 6 software (Graph Pad, USA). The Western blots are representative of at least two independent experiments.

## Supplementary information

Supplementary information

## Data Availability

The data generated during and/or analysed during the current study are available from the corresponding author on reasonable request.
