# Supplementary material for: Novel aroylated phenylenediamine compounds enhance antimicrobial defense and maintain airway epithelial barrier integrity
Source: Sci Rep. 2019 May 8;9:7114. doi: 10.1038/s41598-019-43350-z (PMC6506505; doi:10.1038/s41598-019-43350-z)
Supplement: Supplementary file 1 — Supplementary information [file 41598_2019_43350_MOESM1_ESM.pdf]

Supplementary Information for

Novel aroylated phenylenediamine compounds enhance antimicrobial defense and maintain airway epithelial barrier integrity

Iwona T. Myszor[1], Zahida Parveen[1], Håkan Ottosson[2], Peter Bergman[3], Birgitta Agerberth[3], Roger Strömberg[2], Gudmundur H. Gudmundsson[1]

[1] Biomedical Center; University of Iceland; Reykjavik 101; Iceland

[2] Department of Biosciences and Nutrition; Karolinska Institutet; S-14183 Huddinge; Sweden

[3] Department of Laboratory Medicine; Clinical Microbiology; Karolinska Institutet; S-14186 Huddinge; Sweden

Correspondence and requests for materials should be addressed to G. H. G.; address: Biomedical Center, University of Iceland, Vatnsmyrarvegur 16, Reykjavik 101, Iceland; phone: +354 525 5276; email: ghrafn@hi.is

This PDF file includes:

Supplementary Figure S1. Synthesis scheme for the APD HO53 (*N*-(2-aminophenyl)-4-(2-{[1-(2-hydroxyethyl)-1H-1,2,3-triazol-4-yl]methoxy}-acetamido)benzamide)

Supplementary Figure S2. Synthesis scheme for the APD HO56 (*N*-(2-aminophenyl)-4-{[1-(2-hydroxyethyl)-1H-1,2,3-triazol-4-yl]methoxy}benzamide)

Supplementary Methods

Synthesis for the APD HO53 (*N*-(2-aminophenyl)-4-(2-{[1-(2-hydroxyethyl)-1H-1,2,3-triazol-4-yl]methoxy}-acetamido)benzamide)

Synthesis for the APD HO56 (*N*-(2-aminophenyl)-4-{[1-(2-hydroxyethyl)-1H-1,2,3-triazol-4-yl]methoxy}benzamide)

Supplementary Figure S3. Dose dependent induction of the *CAMP* gene in BCi cells by Entinostat, HO53 and HO56

Supplementary Figure S4. Dose dependent induction of the *CAMP* gene in VA10 cells by Entinostat, HO53 and HO56

Supplementary Figure S5. Cytotoxicity and proliferation of BCi cells after 24 h exposure to Entinostat, HO53 and HO56

Supplementary Figure S6. Effect of APDs treatment on reactive oxygen species (ROS) production and inducible nitric oxide synthase (*NOS2*) in BCi cells

Supplementary Figure S7. Expression of cytokines in ALI differentiated BCi cells upon treatment with HO53 and HO56

Supplementary Figure S8. Azithromycin treatment counteracts disruptive effect of *P. aeruginosa* PAO1 conditioned medium in airway epithelium in ALI culture of BCi cells

Supplementary Figure S9. Changes in occludin pattern in control and HO53 treated airway epithelial BCi cells upon PAO1 conditioned medium challenge

Supplementary Figure S10. Display of full-length blots presented in the article.

Supplementary Table S1. List of primers used in this study

Supplementary Table S2. Quantification of lipocalin 2 expression.

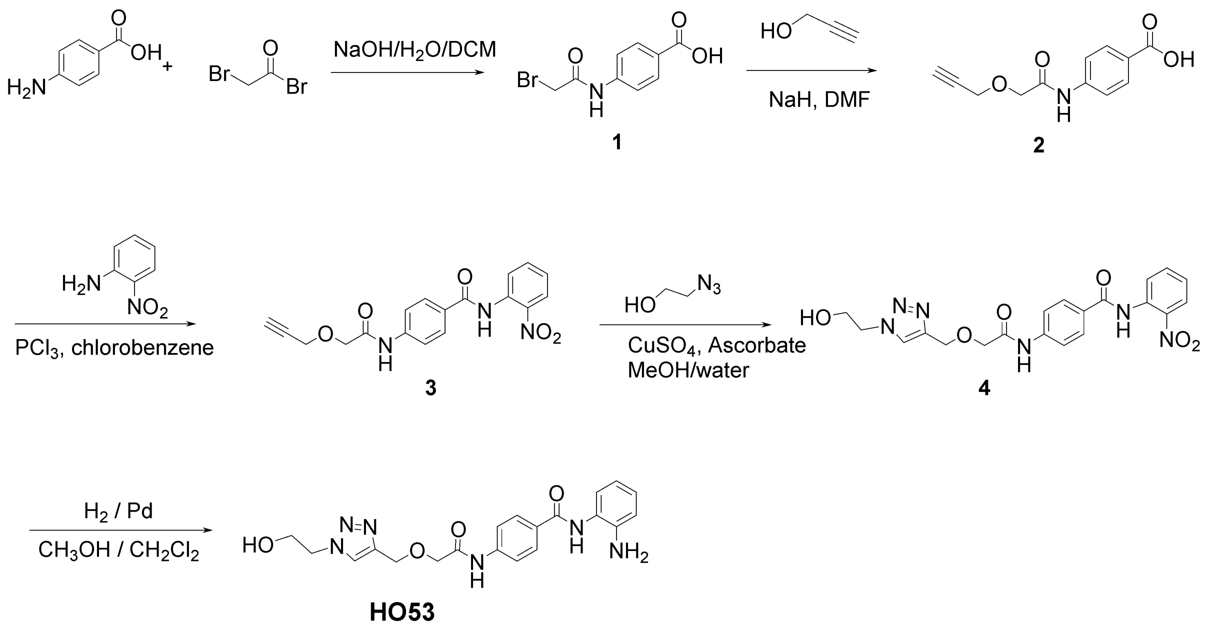

Supplementary Figure S1. Synthesis scheme for the APD HO53 (*N*-(2-aminophenyl)-4-(2-{[1-(2-hydroxyethyl)-1H-1,2,3-triazol-4-yl]methoxy}-acetamido)benzamide). Detailed description of synthesis of the intermediate compounds **1-4** and the final product **HO53** is presented below in Supplementary Methods.

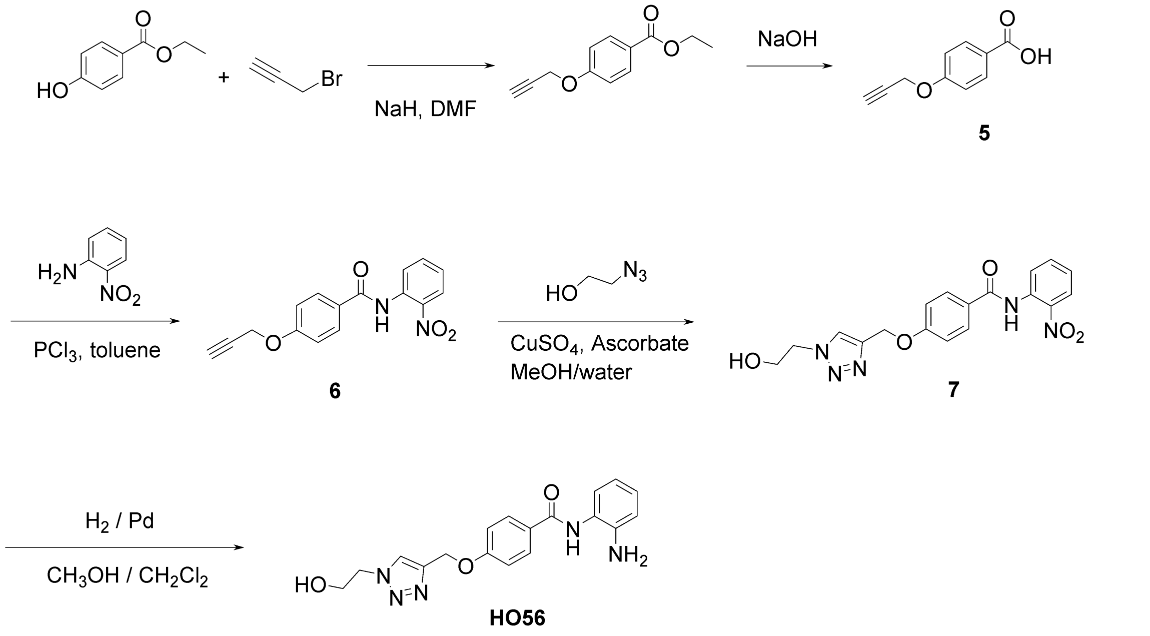

Supplementary Figure S2. Synthesis scheme for the APD HO56 (*N*-(2-aminophenyl)-4-{[1-(2-hydroxyethyl)-1H-1,2,3-triazol-4-yl]methoxy}benzamide). Detailed description of synthesis of the intermediate compounds **5-7** and the final product **HO56** is presented below in Supplementary Methods.

Supplementary Methods

Synthesis for the APD HO53 (*N*-(2-aminophenyl)-4-(2-{[1-(2-hydroxyethyl)-1H-1,2,3-triazol-4-yl]methoxy}-acetamido)benzamide)

*4-(2-bromoacetamido)benzoic acid (**1**).*

Bromoacetyl chloride (5.23 mL, 60.0 mmol) in dichloromethane (15 mL) was added dropwise over 30 min to a solution of 4-aminobenzoic acid (5.49 g, 40.0 mmol) and sodium hydroxide (3.59 g, 89.8 mmol) in water (90 mL) on an ice-salt bath. The precipitate formed was filtered off after ca 3 h (pH ~5). The collected precipitate was washed with water, methanol and dried under reduced pressure. A second precipitate was collected after the filtrate was left overnight and 2.4 mL HCl (conc. *aq*) was added. The second precipitate was washed with water and dried under reduced pressure. The two collected precipitates amounted to 9.3 g (90%) of product which was used as is in the next step. ^{1}H NMR (400 MHz, DMSO-d6) δ ppm: 11.92 - 13.67 (m, 1 H), 10.67 (s, 1 H), 7.92 (d, J=9.06 Hz, 2 H), 7.70 (d, J=8.56 Hz, 2 H), 4.07 (s, 2 H). ^{13}C NMR (101 MHz, DMSO-d6) δ ppm: 166.83, 165.30, 142.57, 130.49, 130.49, 125.74, 118.57, 118.57, 30.29.

*4-{2-[(prop-2-yn-1-yl)oxy]acetamido}benzoic acid (**2**).*

Sodium hydride (60% suspension in oil, 3.25 g, 81.2 mmol) was weighed into a flask which was kept under nitrogen. Dry DMF (5 mL) was added. Propargyl alcohol (5.83 mL, 100 mmol) and 4-(2-bromoacetamido) benzoic acid (5.164 g, 20.0 mmol) in dry DMF (40 mL) was added dropwise at 0 °C. As the reaction got thicker more DMF was added in several portions to a total volume of 250 mL. The reaction was then left at room temperature overnight. The reaction mixture was diluted with dichloromethane. Water was added as well as HCl (6 mL, conc. *aq*). After partitioning additional extraction of the water phase with dichloromethane was done. The combined organic layers were collected pooled and dried over sodium sulfate. The drying agent was filtered off and the solvent was evaporated under reduced pressure to give 4.2 g (90%) of product after drying and that could be used directly in the next step. ^{1}H NMR (400 MHz, DMSO-d6) δ ppm: 7.89 (m, J=8.56 Hz, 2 H), 7.76 (m, J=9.06 Hz, 2 H), 4.27 - 4.33 (m, 2 H), 4.14 - 4.19 (m, 2 H), 3.53 (t, J=2.27 Hz, 1 H), 10.12 (s, 1 H). ^{13}C NMR (101 MHz, DMSO-d6) δ ppm: 168.06, 166.89, 142.46, 130.29, 130.29, 125.46, 118.90, 118.90, 79.57, 78.04, 68.46, 57.92.

*N-(2-nitrophenyl)-4-{2-[(prop-2-yn-1-yl)oxy]acetamido}benzamide (**3**).*

4-{2-[(prop-2-yn-1-yl)oxy]acetamido}benzoic acid (174 mg, 0.746 mmol) and 2-nitroaniline (156 mg, 1.132 mmol) was dissolved in chlorobenzene (10 mL). PCl_3 (33 µL, 0.378 mmol) was added and the reaction was heated for 2 h at 130 °C and then left at room temperature overnight. The mixture was diluted with dichloromethane and water was added. After partitioning the organic layer was washed with 0-1 M HCl and dried with sodium sulfate. After removal of the solvent under reduced pressure the residue was recrystallized from ethanol (two crops) to yield the product as a light yellow solid (133 mg, 50%). ^{1}H NMR (400 MHz, DMSO-d6) δ ppm: 10.68 (s, 1 H), 10.17 (s, 1 H), 8.01 (dd, J=8.10, 1.51 Hz, 1 H), 7.94 (d, J=8.56 Hz, 2 H), 7.78 - 7.86 (m, 3 H), 7.75 (td, J=7.80, 1.51 Hz, 1 H), 7.41 (ddd, J=8.50, 7.00, 1.50 Hz, 1 H), 4.32 (d, J=2.52 Hz, 2 H), 4.16 - 4.21 (m, 2 H), 3.56 (t, J=2.52 Hz, 1 H). ^{13}C NMR (101 MHz, DMSO-d6) δ ppm: 168.13, 164.70, 142.06, 134.05, 131.77, 128.72, 128.72, 125.77, 125.36, 125.00, 119.05, 119.05, 79.60, 78.11, 68.48, 57.97.

*4-(2-{[1-(2-hydroxyethyl)-1H-1,2,3-triazol-4-yl]methoxy}acetamido)-N-(2-nitrophenyl)-benzamide (**4**).*

N-(2-nitrophenyl)-4-{2-[(prop-2-yn-1-yl)oxy]acetamido}benzamide (71 mg, 0.201 mmol) and 2-azidoethanol (73 µL, 1.00 mmol) was mixed with methanol (46 mL) and water (4 mL). the mixture was then heated to dissolve the reagents. $CuSO_4*5H_2O$ (50 mg, 0.200 mmol) and ascorbic acid (142 mg, 0.800 mmol) were added while the mixture was still warm. The mixture became cloudy after a few minutes and was kept overnight at room temperature. EDTA (95 mg) was added and the mixture was then diluted with dichloromethane. Partitioning of the dichloromethane solution with water (with approx. 1/5 brine). The dichloromethane phase was washed with water containing 1/5 brine and EDTA (ca 100 mg). The collected organic layer was dried with Na_2SO_4, filtered and concentrated under reduced pressure. The resulting yellow solid was recrystallized from ethanol to yield 59 mg (67 %) of product as a yellow solid. ^{1}H NMR (400 MHz, DMSO-d6) δ ppm: 10.68 (s, 1 H), 10.14 (s, 1 H), 8.15 (s, 1 H), 8.01 (dd, J=8.06, 1.51 Hz, 1 H), 7.89 - 7.98 (m, 2 H), 7.84 (d, J=8.56 Hz, 2 H), 7.80 (d, J=8.06 Hz, 1 H), 7.75 (ddd, J=8.20, 7.00, 1.50 Hz, 1 H), 7.41 (ddd, J=8.10, 7.00, 1.51 Hz, 1 H), 5.05 (t, J=5.29 Hz, 1 H), 4.70 (s, 2 H), 4.41 (t, J=5.40 Hz, 2 H), 4.17 (s, 2 H), 3.78 (q, J=5.40 Hz, 2 H). ^{13}C NMR (101 MHz, DMSO-d6) δ ppm: 168.55, 164.69, 143.04, 142.71, 142.07, 134.04, 131.76, 128.70, 128.70, 128.10, 125.77, 125.37, 124.99, 124.74, 119.03, 119.03, 69.13, 63.82, 59.87, 52.18.

N-(2-aminophenyl)-4-(2-{[1-(2-hydroxyethyl)-1H-1,2,3-triazol-4-yl]methoxy}-acetamido)-benzamide (**HO53**).

To a solution of 4-(2-{[1-(2-hydroxyethyl)-1H-1,2,3-triazol-4-yl]methoxy}acetamido)-N-(2-nitrophenyl)benzamide (39 mg, 0.0886 mmol) in MeOH (4 mL) and DCM (4 mL), activated palladium on carbon (~10% by weight, 21 mg) was added. The flask was fitted with a rubber septum and the mixture was flushed with N_2 (g) via a needle twice. A balloon filled with H_2 (g) was connected and the mixture was stirred for 2.5 h at room temperature. After flushing, with nitrogen the solution was filtered through silica and evaporated under reduced pressure to give 31 mg (86 %) of product. 1H NMR (400 MHz, MeOH) δ ppm: 8.07 (s, 1 H), 7.97 (m, J=8.56 Hz, 2 H), 7.79 (m, J=8.56 Hz, 2 H), 7.16 - 7.21 (m, 1 H), 7.04 - 7.11 (m, 1 H), 6.90 (d, J=7.55 Hz, 1 H), 6.77 (t, J=7.55 Hz, 1 H), 4.78 (s, 2 H), 4.50 (t, J=5.04 Hz, 2 H), 4.11 - 4.21 (m, 2 H), 3.93 (t, J=5.29 Hz, 2 H). 13C NMR (101 MHz, DMSO-d6) δ ppm: 170.86, 168.34, 144.94, 143.96, 142.58, 131.14, 129.89, 129.89, 128.67. MS (ESI-TOF) m/z: $[M^{-}H]^{-}$: calcd. for $C_{20}H_{21}N_6O_4$, 409.2, found, 409.1. m/z: $[M^{+}H]^{+}$: calcd. for $C_{20}H_{23}N_6O_4$, 411.2, found, 411.2.

Synthesis for the APD HO56 (*N*-(2-aminophenyl)-4-{[1-(2-hydroxyethyl)-1H-1,2,3-triazol-4-yl]methoxy}benzamide).

*4-[(prop-2-yn-1-yl)oxy] benzoic acid (**5**).*

Ethyl 4-hydroxybenzoate (8.308 g, 50.0 mmol) in DMF (20 mL) was added dropwise to sodium hydride (2.278 g, 57.0 mmol) in DMF (15 mL) at 0 °C followed by dropwise addition of propargyl bromide (80% in toluene, 6.465 mL, 60.0 mmol) in DMF (10 mL). After reaching room temperature the reaction mixture was left stirring overnight. Water (10 mL) was added and then sodium hydroxide (2.15 g, 53.7 mmol). The mixture was washed with dichloromethane and the water layer was acidified with HCl to give a precipitate that after drying under reduced pressure amounted to 3.28 g (37%) of the desired product. ^{1}H NMR (400 MHz, DMSO-d6) δ ppm: 7.91 (d, J=8.56 Hz, 2 H), 7.07 (d, J=8.56 Hz, 2 H), 4.88 (d, J=2.52 Hz, 2 H), 3.60 (t, J=2.27 Hz, 1 H). ^{13}C NMR (101 MHz, DMSO-d6) δ ppm: 166.92, 160.74, 131.28, 131.28, 123.70, 114.67, 114.67, 78.76, 78.64, 55.66.

*N-(2-nitrophenyl)-4-[(prop-2-yn-1-yl)oxy]benzamide (**6**).*

A mixture of 4-[(prop-2-yn-1-yl)oxy] benzoic acid (353 mg, 2.0 mmol) and 2-nitroaniline (415 mg, 3.0 mmol) in toluene (30 mL) was heated to boiling. After some cooling, phosphorus trichloride (87 µL, 1.0 mmol) was added. The mixture was reflux for 2 h, cooled to room temperature, diluted with dichloromethane. Partitioning was the done between the dichloromethane solution and an added 0.1 M HCl (*aq*) phase. The organic phase was washed with 0.1 M HCl, water and aqueous sodium bicarbonate. The organic layer then collected,

dried with sodium sulfate, and concentrated under reduced pressure. The resulting solid was recrystallized from ethanol to give a yellow powder (365 mg, 61%). ^{1}H NMR (400 MHz, DMSO-d6) δ ppm: 10.66 (s, 1 H), 8.01 (dd, J=8.31, 1.26 Hz, 1 H), 7.95 (d, J=9.06 Hz, 1 H), 7.78 (td, J=8.06, 1.51 Hz, 1 H), 7.73 (dd, J=8.06, 1.50 Hz, 1 H), 7.40 (ddd, J=8.06, 7.05, 1.51 Hz, 1 H), 7.15 (d, J=9.06 Hz, 1 H), 4.92 (d, J=2.52 Hz, 1 H), 3.63 (t, J=2.52 Hz, 1 H). ^{13}C NMR (101 MHz, DMSO-d6) δ ppm: 164.77, 160.34, 142.78, 134.10, 131.83, 129.74, 129.74, 126.36, 125.83, 125.40, 125.05, 114.86, 114.86, 78.84, 78.76, 55.74.

*4-{[1-(2-hydroxyoethyl)-1H-1,2,3-triazol-4-yl]methoxy}-N-(2-nitrophenyl)benzamide (**7**).*
N-(2-nitrophenyl)-4-[(prop-2-yn-1-yl)oxy]benzamide (503.6 mg, 1.70 mmol) and 2-azidoethanol (370 µL, 5.10 mmol) was mixed with methanol (225 mL) and water (25 mL). The mixture was then heated to dissolve the reagents. $CuSO_4$*5H_2O (213 mg, 0.85 mmol) and ascorbic acid (1.20 g, 6.82 mmol) were added at 50 - 60 ^oC. The reaction was left overnight at room temperature. EDTA was added and the mixture was then diluted with dichloromethane. Partitioning of the dichloromethane solution with water (with approx. 1/5 brine). The dichloromethane phase was washed with water containing 1/5 brine and EDTA (ca 100 mg). The water layer was extracted with ethyl acetate and the collected organic layers were dried with Na_2SO_4, filtered and concentrated under reduced pressure. The resulting yellow solid was recrystallized from ethanol to yield 364 mg (56 %) of product as a yellow solid. ^{1}H NMR (400 MHz, DMSO-d6) δ ppm 10.64 (s, 3 H), 8.24 (s, 4 H), 8.01 (dd, J=8.06, 1.51 Hz, 1 H), 7.92 - 7.98 (m, 2 H), 7.81 (dd, J=8.31, 1.26 Hz, 1 H), 7.75 (td, J=7.55, 1.50 Hz, 1 H), 7.40 (ddd, J=8.00, 7.80, 1.50 Hz, 1 H), 7.22 (d, J=8.56 Hz, 8 H), 5.26 (s, 2 H), 5.07 (t, J=5.29 Hz, 3 H), 4.42 (t, J=5.29 Hz, 8 H), 3.79 (q, J=5.54 Hz, 8 H). ^{13}C NMR (101 MHz, DMSO-d6) δ ppm 165.27, 161.62, 142.90, 136.34, 136.08, 129.31, 129.31, 126.53, 125.81, 124.33, 123.17, 122.03, 121.95, 114.91, 114.91, 61.73, 60.46, 52.69.

N-(2-aminophenyl)-4-{[1-(2-hydroxyethyl)-1H-1,2,3-triazol-4-yl]methoxy}benzamide (**HO56**).
To a solution of N-(2-aminophenyl)-4-{[1-(2-hydroxyethyl)-1H-1,2,3-triazol-4-yl]methoxy}benzamide (23.9 mg, 0.062 mmol) in methanol (10 mL) and dichloromethane (5 mL), activated palladium on carbon (~10% by weight, 10 mg) was added. The flask was fitted with a rubber septum and the mixture was flushed with N_2 (g) via a needle twice. A balloon of H2 gas was introduced at room temperature with stirring. A balloon filled with H_2 (g) was connected and the mixture was stirred for 2 h at room temperature. After flushing with nitrogen the solution was filtered through Celite and evaporated under reduced pressure to give an off-

white powder (21 mg, 0,060 mmol, 96%). ^{1}H NMR (400 MHz, DMSO-d6) δ ppm: 9.57 (s, 1 H), 8.23 (s, 1 H), 7.97 (d, J=8.06 Hz, 2 H), 7.15 (d, J=7.05 Hz, 4 H), 6.96 (s, 1 H), 6.77 (d, J=8.06 Hz, 1 H), 6.59 (s, 1 H), 5.23 (s, 2 H), 5.08 (t, J=5.04 Hz, 1 H), 4.88 (br. s., 2 H), 4.42 (t, J=5.29 Hz, 2 H), 3.75 - 3.84 (m, 2 H). ^{13}C NMR (101 MHz, DMSO-d6) δ ppm: 164.70, 160.55, 143.21, 142.05, 129.67, 129.67, 127.00, 126.74, 126.36, 125.19, 123.54, 116.26, 116.14, 114.21, 114.21, 61.23, 59.83, 52.25. MS (ESI-TOF) m/z: $[M^{-}H]^{-}$: calcd. for $C_{20}H_{21}N_6O_4$, 252.1, found, 252.0. m/z: $[M^{+}H]^{+}$: calcd. for $C_{20}H_{23}N_6O_4$, 354.16, found, 354.1.

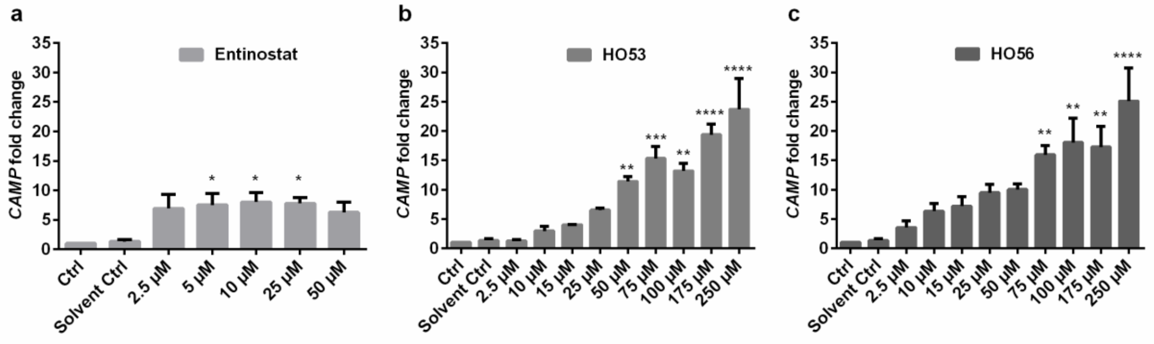

Supplementary Figure S3. Dose dependent induction of the *CAMP* gene in BCi cells by Entinostat, HO53 and HO56. Cells were stimulated for 24 h with increasing doses of **a)** Entinostat, **b)** HO53 and **c)** HO56. DMSO (final concentration lower than 1%) was used as a solvent control (Solvent Ctrl). The *CAMP* gene expression was normalized to *TUBB* (tubulin-β) reference gene and presented as fold change of the expression in comparison to control cells (Ctrl). Each bar represents mean value of 3 independent experiments ± SEM; statistical significance was calculated in comparison to control cells using one-way ANOVA with Dunnett´s multiple comparisons test; * $p<0.05$, ** $p<0.01$, *** $p<0.001$, **** $p<0.0001$. All tested concentrations are shown and selected concentrations are presented in figure 1b.

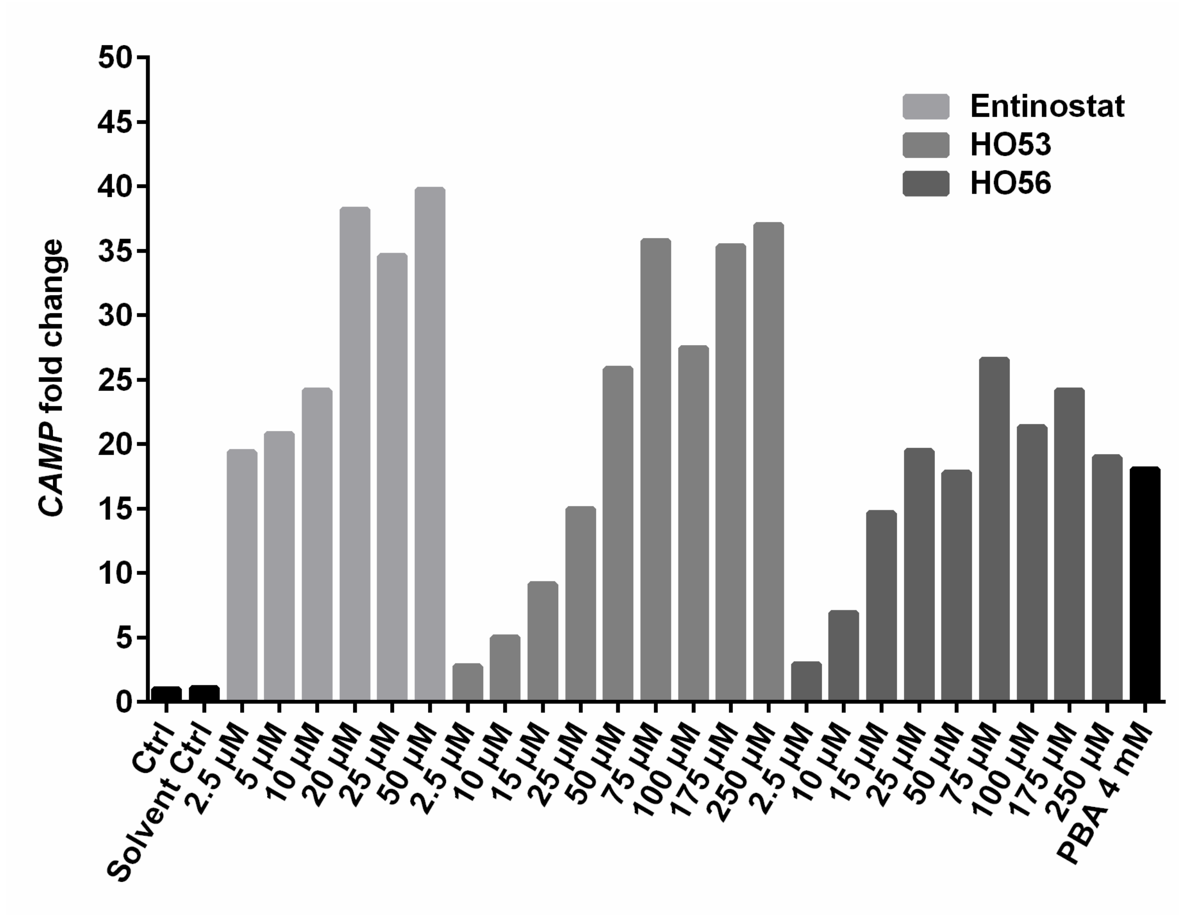

Supplementary Figure S4. Dose dependent induction of the *CAMP* gene in VA10 cells by Entinostat, HO53 and HO56. Cells were stimulated for 24 h with increasing doses of Entinostat (2.5-50 µM), HO53 and HO56 (both at 2.5-250 µM). DMSO (final concentration lower than 1%) and PBA (4 mM) were used as a solvent control (Solvent Ctrl) and positive control, respectively. The *CAMP* gene expression was normalised to *TUBB* (tubulin-β) reference gene and presented as fold change of the expression in comparison to control cells (Ctrl). Each bar is representative of technical duplicates.

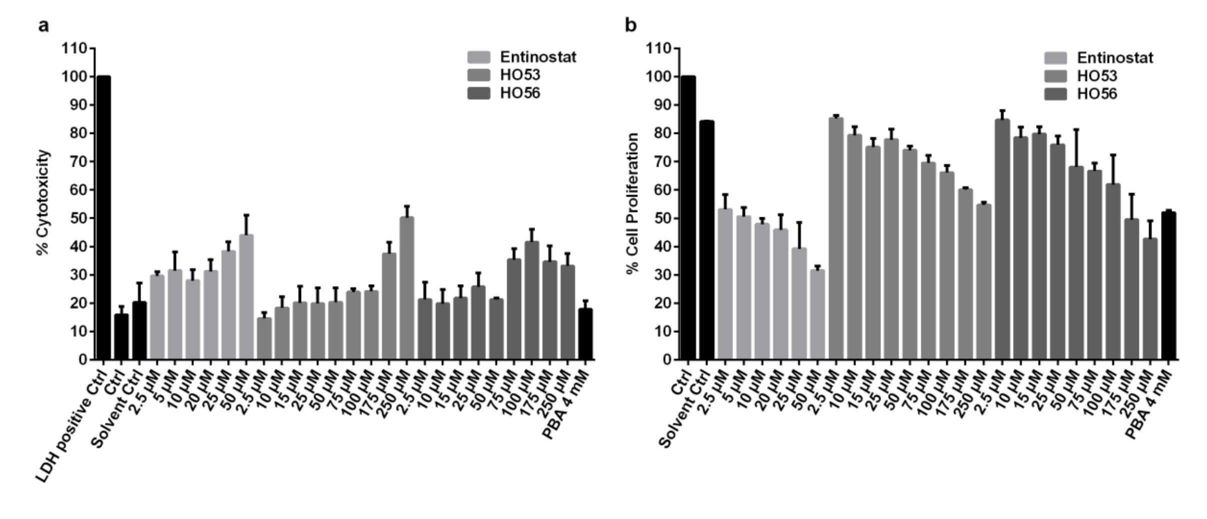

Supplementary Figure S5. Cytotoxicity and proliferation of BCi cells after 24 h exposure to Entinostat, HO53 and HO56. a) Cytotoxic effect of Entinostat (2.5-50 µM), HO53 (2.5-250 µM) and HO56 (2.5-250 µM) on BCi cells was determined by the LDH assay. Cytotoxicity was measured as a percentage of the LDH positive control. Each bar is the mean value of 3 independent experiments ± SEM. Using unpaired student t-test *p value* of selected concentration 75 µM HO53 and HO56 in comparison to solvent control is 0.237 and 0.027, respectively. **b)** Effect of Entinostat, HO53 and HO56 on cell proliferation was determined by WST-1 assay. Data shown as percentage of the control cells and presented as a mean value of 2 independent experiments ± SEM. Using unpaired student t-test *p value* of selected concentration 75 µM HO53 and HO56 in comparison to solvent control is 0.0437 and 0. 0336, respectively.

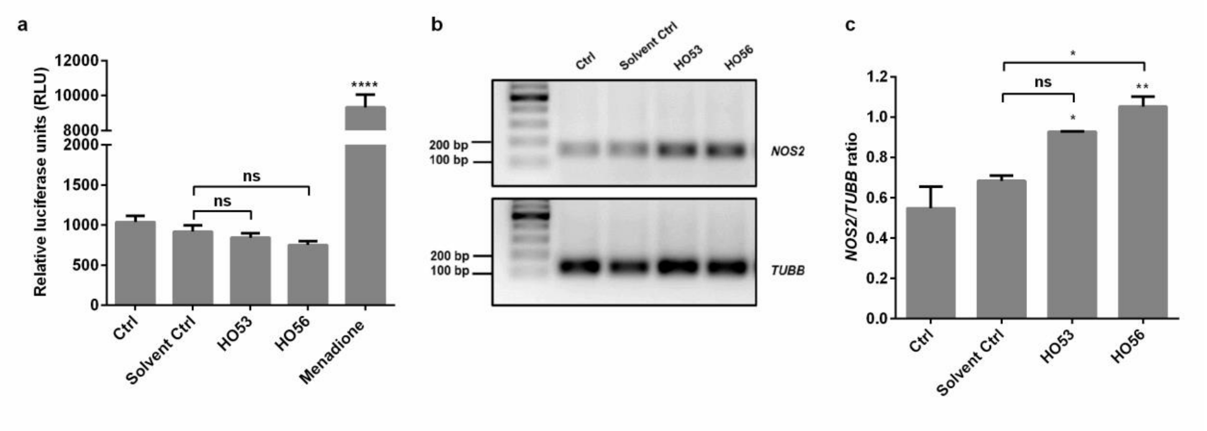

Supplementary Figure S6. Effect of HO53 and HO56 treatment on reactive oxygen species (ROS) production and inducible nitric oxide synthase (*NOS2*) in BCi cells. a) The H_2O_2 level in cell culture medium was measured in relative luciferase units (RLU) after 24 h of induction with HO53 and HO56 (both at 75 µM). Menadione (50 µM) treatment for 90 min served as a positive control. Data is representative of n=3 independent experiments ± SEM, statistical significance was calculated using one-way ANOVA with Dunnett´s multiple comparisons test while comparing to control (Ctrl) or with Sidak´s multiple comparisons test while comparing HO53 and HO56 to solvent control (Solvent Ctrl). **b)** *NOS2* expression accessed by semi-quantitative PCR (representative of 2 independent experiments). **c)** Quantification of *NOS2/TUBB* ratio of 2 independent experiments ± SEM, statistical significance was calculated using one-way ANOVA with Dunnett´s multiple comparisons test while comparing to control (Ctrl) or with Sidak´s multiple comparisons test while comparing HO53 and HO56 to solvent control (Solvent Ctrl). * $p<0.05$, ** $p<0.01$, **** $p<0.0001$, ns indicates non-significant.

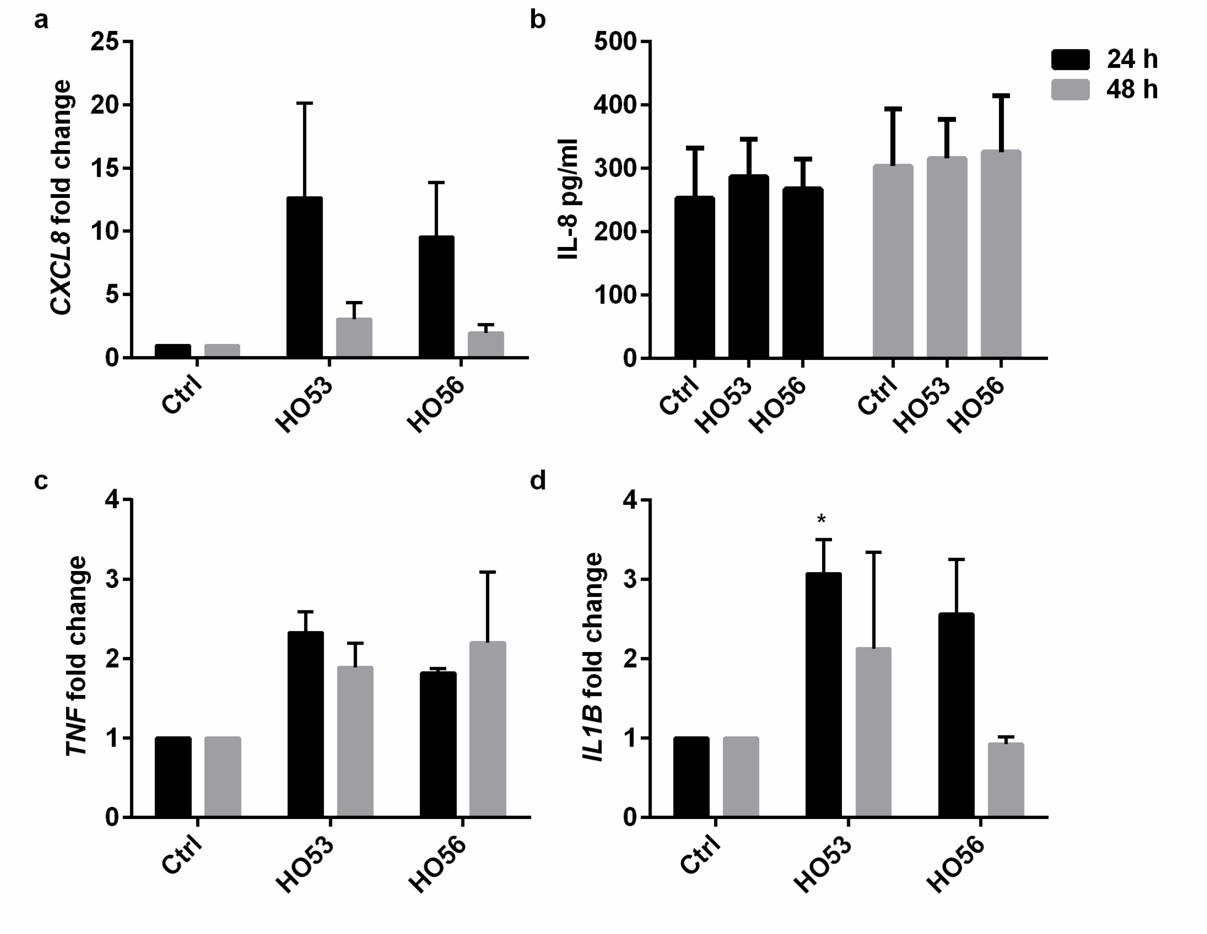

Supplementary Figure S7. Expression of cytokines in ALI differentiated BCi cells upon treatment with HO53 and HO56. mRNA expression of **a)** *CXCL8*, **c)** *TNF* and **d)** *IL1B* measured by qRT-PCR and normalized to *TUBB* (tubulin-β) reference gene. Data is from n=4 independent experiments ± SEM. **b)** Protein level of IL-8 was accessed by ELISA. Data is representative of n=2 independent experiments ± SEM. Statistical significance was calculated in comparison to untreated cells (Ctrl) using two-way ANOVA with Dunnett´s multiple comparisons test, * $p<0.05$, only significant changes were highlighted.

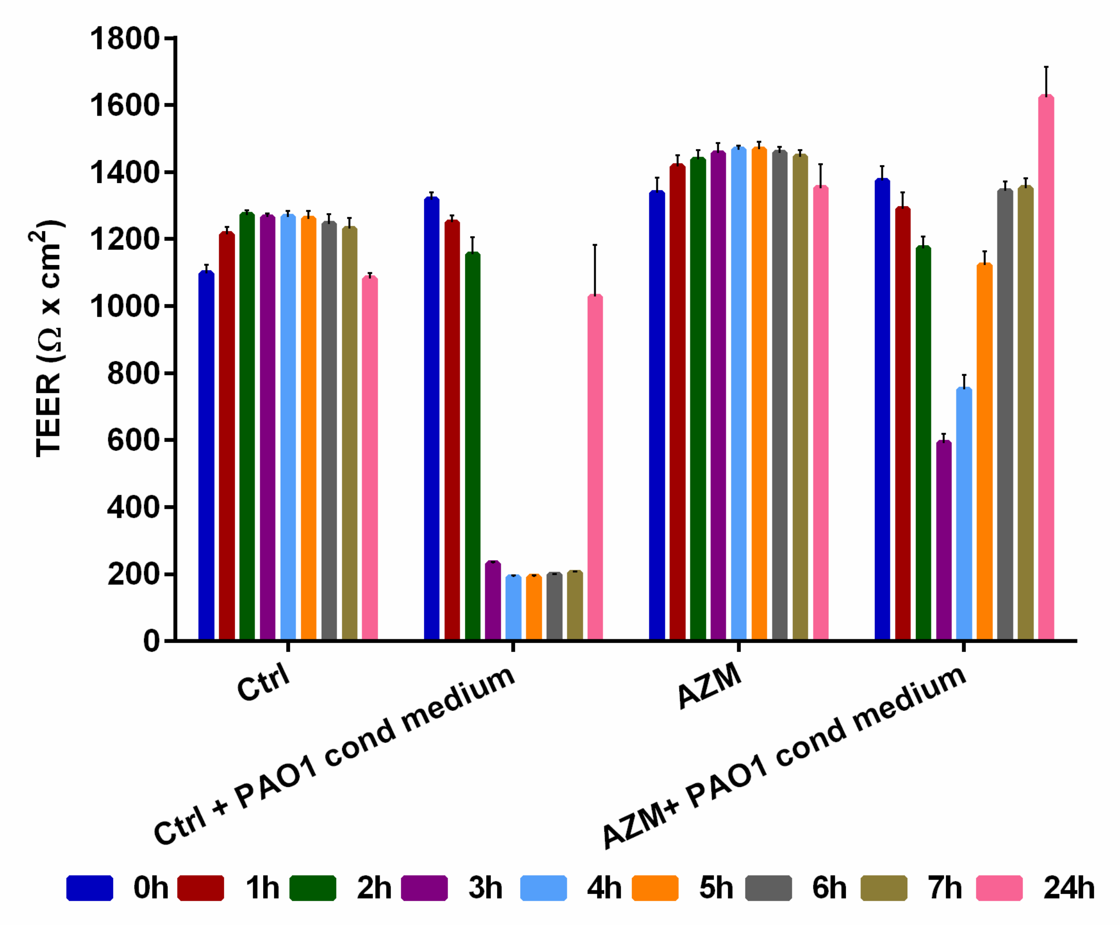

Supplementary Figure S8. Azithromycin treatment counteracts disruptive effect of *P. aeruginosa* PAO1 conditioned medium in airway epithelium in ALI culture of BCi cells. Differentiated BCi cells (TEER ≥1000 Ω×cm^2) were treated with 40 µg/mL Azithromycin in the lower chamber of transwell insert for 3 days and challenged with PAO1 conditioned medium applied on the apical surface of the cells. TEER (Trans epithelial electrical resistance) measurement after every hour from 0 to 7 h and after 24 h post PAO1 culture medium challenge with and without Azithromycin basal treatment. Data shown is representative of two experiments, showing mean values of four independent ALI filters at each time point ± SEM.

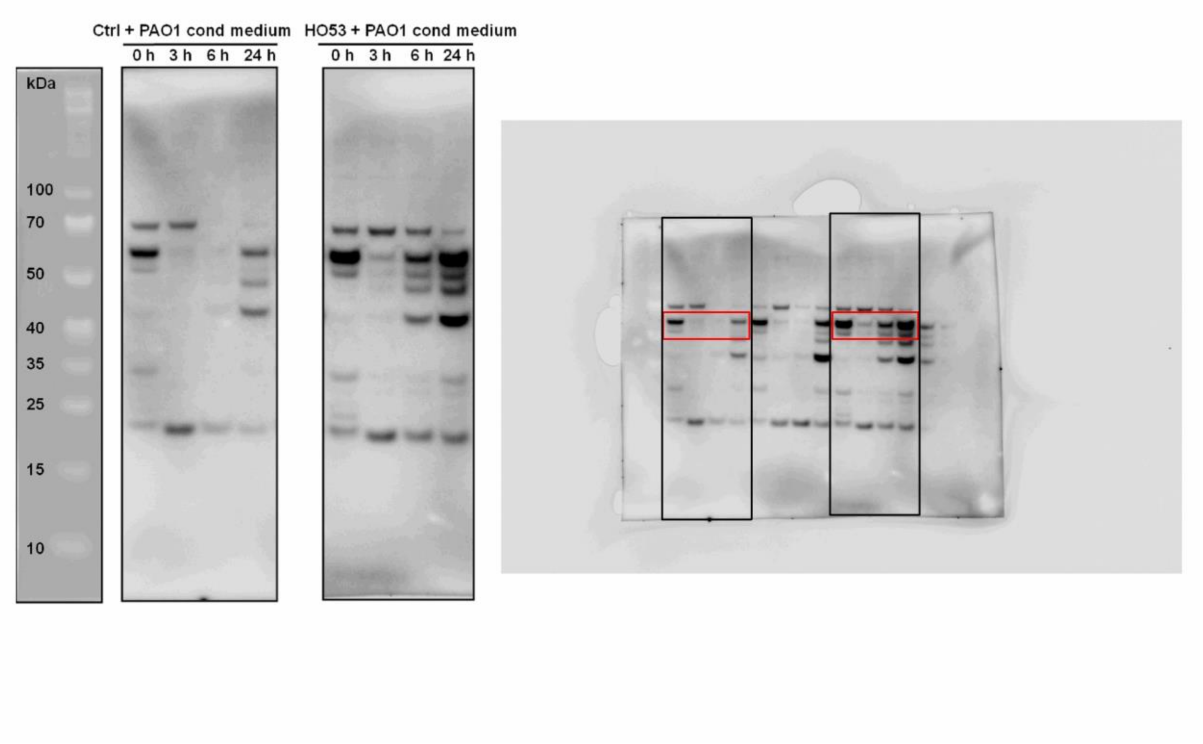

Supplementary Figure S9. Changes in occludin pattern in control and HO53 treated airway epithelial BCi cells upon PAO1 conditioned medium challenge. The main occludin isoform is the ~ 60 kDa band and it is presented in Figure 6c. The uncropped immunoblot is presented on the right.

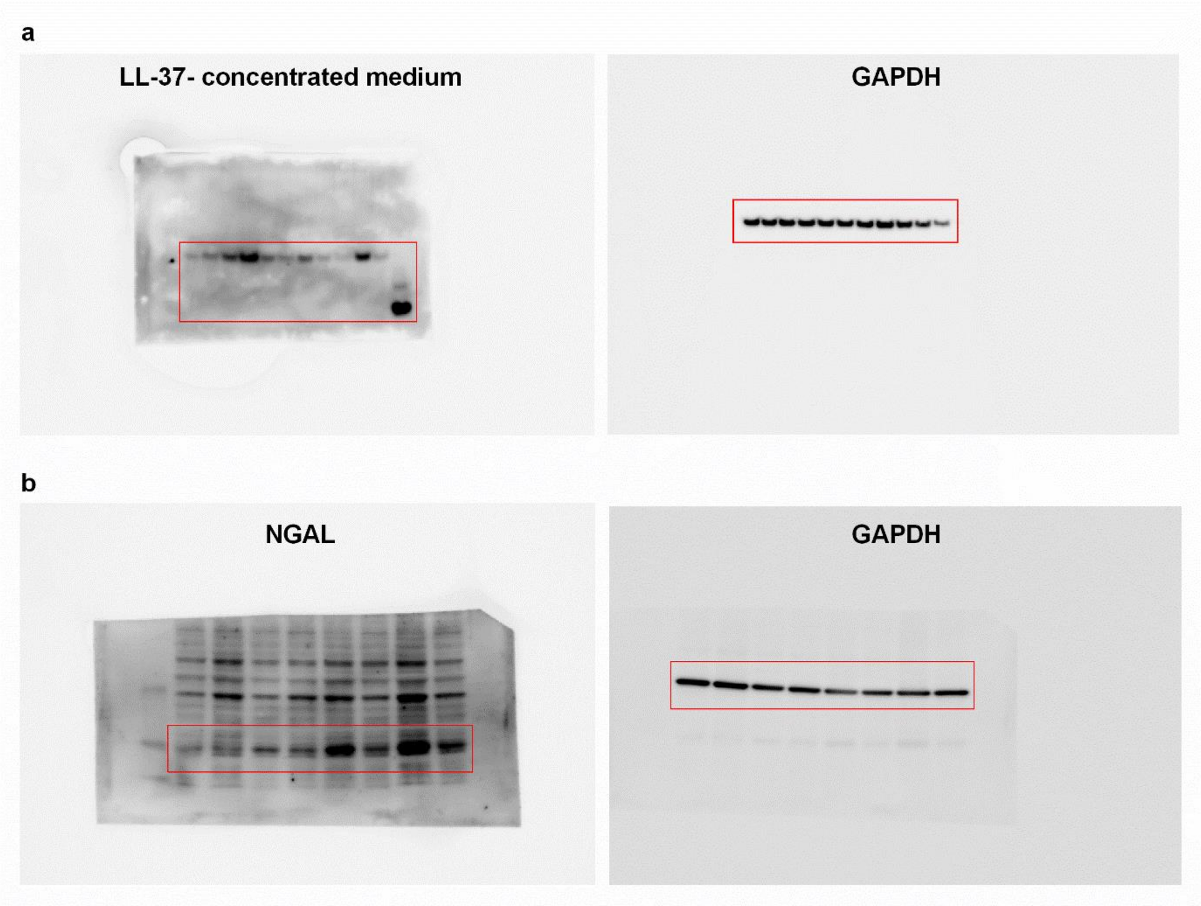
a
LL-37- concentrated medium
GAPDH
b
NGAL
GAPDH

c

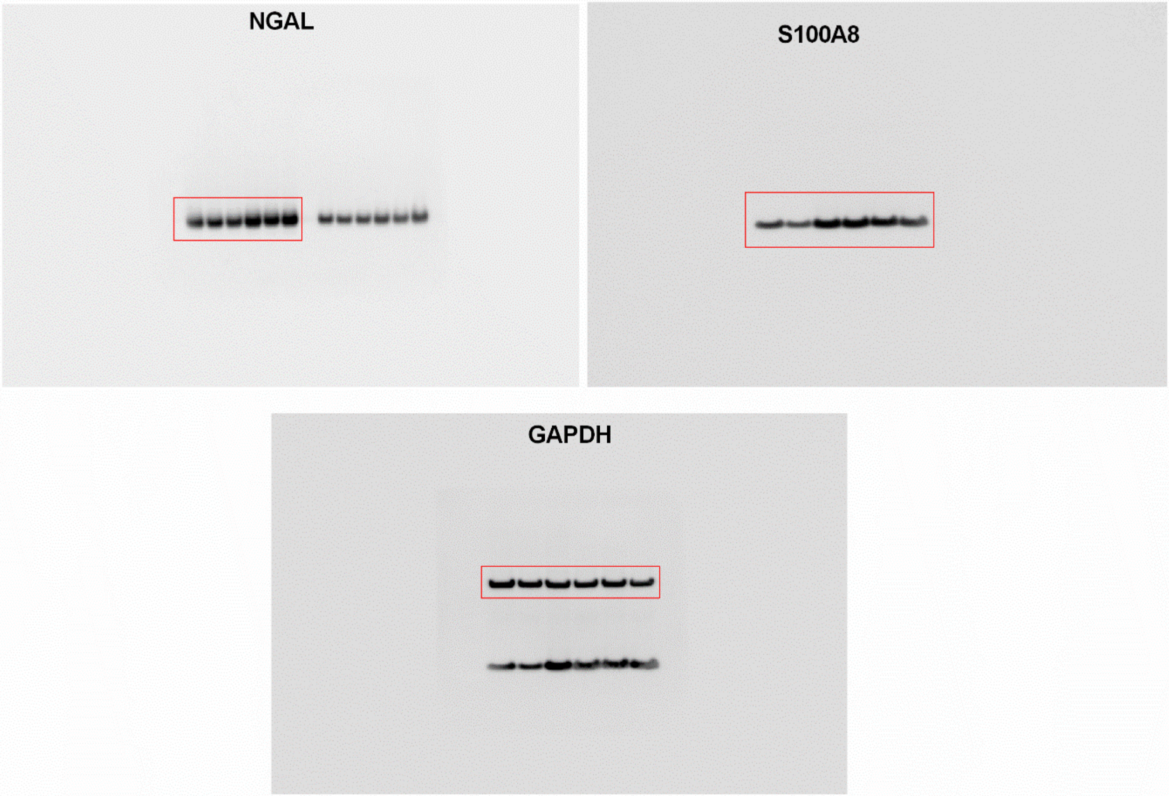

d

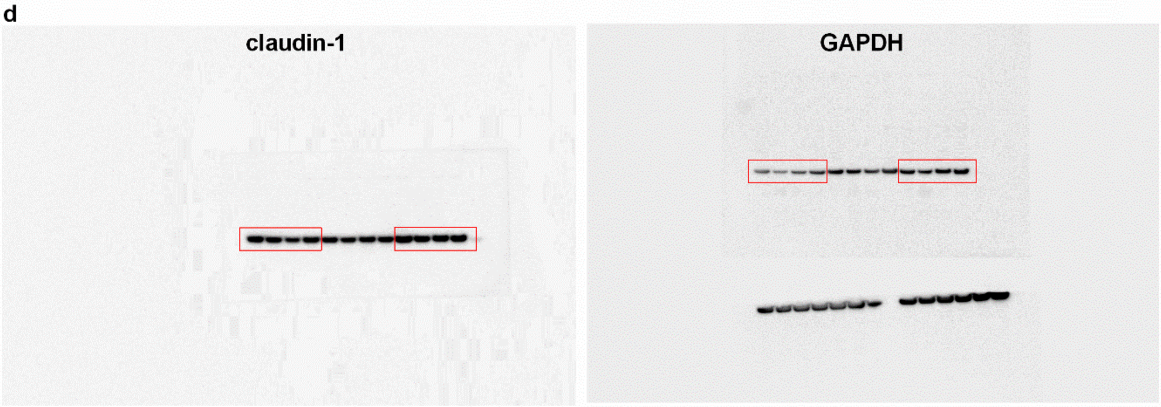

e

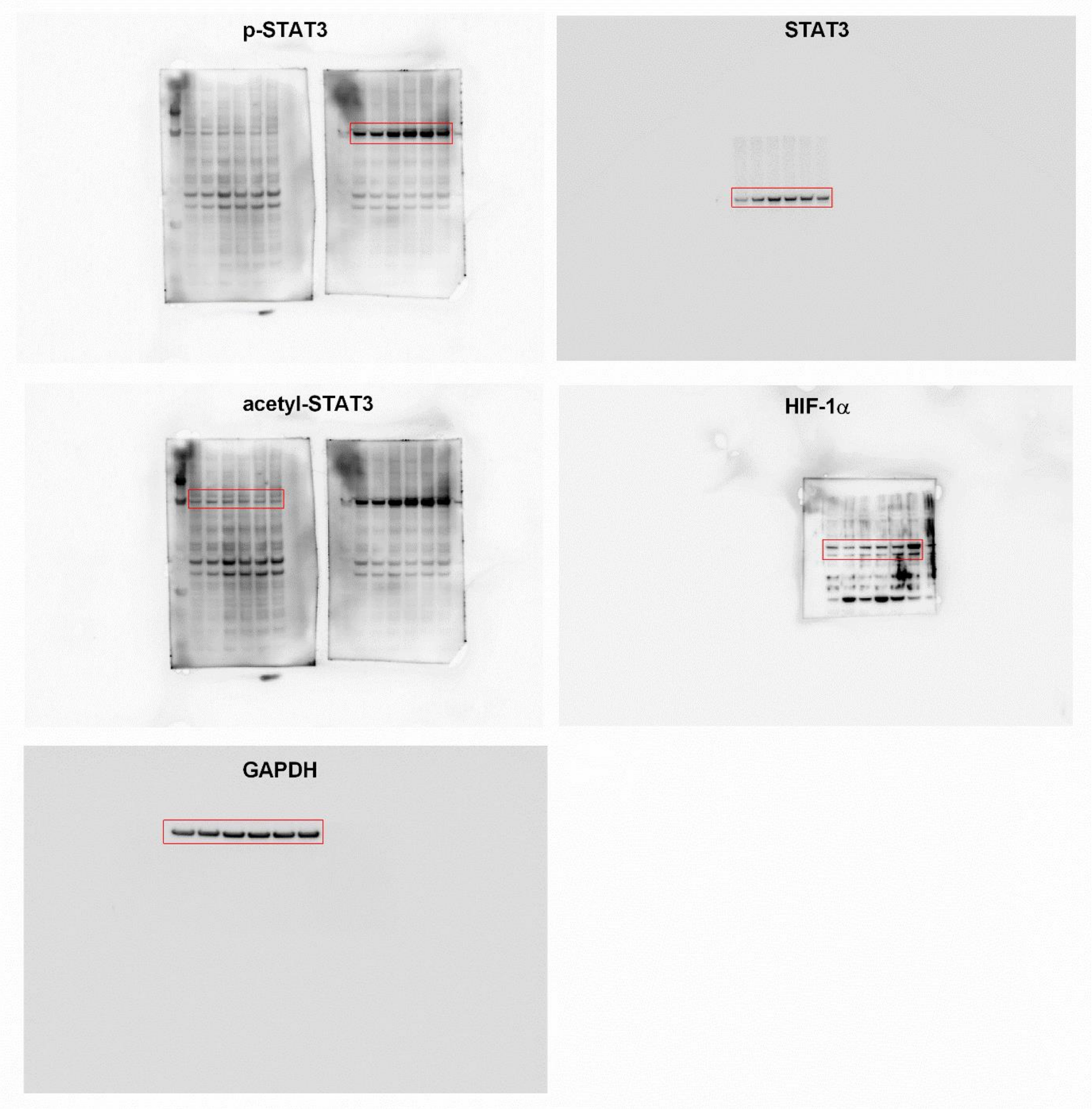

Supplementary Figure S10. Display of full-length blots presented in the article. Uncropped immunoblots showing: **a)** pro-LL-37 in the concentrated medium and synthetic LL-37 together with GAPDH loading control (presented in Fig. 1g), **b)** NGAL and GAPDH (presented in Fig. 2b), **c)** NGAL, S100A8 and GAPDH (presented in Fig. 5c), **d)** claudin-1 and GAPDH (presented in Fig. 6c), **e)** p-STAT3, STAT3, acetyl-STAT3, HIF-1α, GAPDH (presented in Fig. 7c).

Supplementary Table S1. List of primers used in this study

Gene	RefSeq number	Forward Primer (5´→3´)	Reverse Primer (5´→3´)
CAMP	NM_004345.4	GCACACTGTCTCCTTCACTG	CTAACCTCTACCGCCTCCT
HBD1	NM_005218.3	CCAGTCGCCATGAGAACTTCC	GTGAGAAAGTTACCACCTGAGGC
LCN2	NM_005564.4	AACTTCATCCGCTTCTCCAA	TCTCCCAGCTCCCTCAATG
LYZ	NM_000239.2	AGATAACATCGCTGATGCTGTAG	CTCCACAACCTTGAACATACTGA
LTF	NM_001321122.1	TGTATCCAGGCCATTGCG	ATAGTGAGTTCGTGGCTGTC
S100A8	NM_002964.4	TCTACCACAAGTACTCCCTGAT	TCCAACTCTTTGAACCAGACG
CXCL8	NM_000584.3	CTGTCTGGACCCCAAGGAA	CTGGCATCTTCACTGATTCTTG
IL1B	NM_000576.2	CAGCCAATCTTCATTGCTCAAG	GAACAAGTCATCCTCATTGCC
TNF	NM_000594.3	CCTCTCTCTAATCAGCCCTC	CCTCAGCTTGAGGGTTTGC
NOS2	NM_000625.4	AATGAATACCGGTCCCGTGG	CATGGGTTTTCCAGGCCTCT
HIF1A	NM_001530	CCATTAGAAAGCAGTTCCGC	TGGGTAGGAGATGGAGATGC
TUBB	NM_178014.3	GCCAGATCTTTAGACCAGACAA	CCTCCTTCCGTACCACATC

Supplementary Table S2. Quantification of lipocalin 2 expression. The representative relative quantification of NGAL/GAPDH ratio normalized to control from Western blotting presented in Fig. 2b.

Lane	Treatment	NGAL	GAPDH	NGAL/GAPDH ratio	Relative NGAL/GAPDH ratio normalized to control
1	ctrl-24h	8336.347	36005.47	0.23153	1.00
2	ctrl-48h	14835.2	38151.83	0.388846	1.00
3	Entinostat-24h	10763.47	25078.3	0.429195	1.85
4	Entinostat-48h	15800.42	28032.13	0.563654	1.45
5	HO53-24h	31168	20517	1.51913	6.56
6	HO53-48h	20721.81	23823	0.869824	2.24
7	HO56-24h	38882.83	24053.71	1.6165	6.98
8	HO56-48h	24374.05	28127.25	0.866564	2.23
